# Comparative resistome from toilet waste in three different income areas, Bangkok, Thailand

**DOI:** 10.3389/fmicb.2026.1790551

**Published:** 2026-03-25

**Authors:** Narong Nuanmuang, Pimlapas Leekitcharoenphon, Patrick Murigu Kamau Njage, Jiraporn Jirakkakul, Sudarat Dulsawat, Anuwat Tachaleat, Christina Aaby Svendsen, Frederik Duus Møller, Saria Otani, Supapon Cheevadhanarak, Frank M. Aarestrup

**Affiliations:** 1 Research Group for Genomic Epidemiology, National Food Institute, Technical University of Denmark, Kgs. Lyngby, Denmark; 2 Pilot Plant Development and Training Institute, King Mongkut’s University of Technology Thonburi, Bangkok, Thailand; 3 Bioinformatics and Systems Biology Program, School of Bioresources and Technology and School of Information Technology, King Mongkut’s University of Technology Thonburi, Bangkok, Thailand

**Keywords:** antibiotic resistance genes, antimicrobial resistance, metagenomics, resistome, toilet waste

## Abstract

Antimicrobial resistance (AMR) is a significant public health threat and is associated with millions of deaths worldwide each year. Besides antimicrobial usage, different socioeconomic factors have recently gained attention as being associated with increased AMR. Bangkok, a city with diverse income levels, provided a unique setting for this study, which aimed to explore the possible within-city association between income-level areas and the diversity and abundance of AMR. Twenty-seven toilet waste samples were collected from nine different sites (low-, middle-, and high-income) during March–April 2023, and metagenomic sequencing was performed. The sequencing data were quality checked, and sequences that passed quality control were mapped to antimicrobial, metal, and disinfectant resistance gene databases as well as bacterial taxonomy databases. We observed higher antibiotic resistance genes (ARGs), metal resistance, and disinfectant resistance abundance (fragments per kilobase per million mapped reads, FPKM) in low-income groups compared to middle- and high-income groups. This included both acquired ARGs and presumed intrinsic ARGs, including genes associated with completely novel antibiotics that have so far only been identified through functional cloning. Significant differences in individual ARGs were also observed between sites. Our study highlights the relative abundance of ARGs across different income groups, emphasizing how the development of resistance mechanisms revealed through metagenomic analysis can serve as a valuable tool for city-level surveillance of AMR from toilet waste, particularly in low-income settings.

## 1 Introduction

Antimicrobial resistance (AMR) is a global health threat leading to prolonged illness, increased healthcare expenditures, and elevated mortality rates. It has been estimated that 4.95 million deaths were associated with and 1.27 million deaths were attributed to bacterial AMR, respectively (Murray et al., 2022). AMR is associated with antimicrobial use (AMU), which is a key driver of AMR development. However, several contributing factors also affect resistance, including health, nutrition, and socioeconomic status across countries (Hendriksen et al., 2019; Njage et al., 2023).

Surveillance is key to determining the scale of the AMR problem and to understanding the differences between niches and sites. However, sampling from clinically healthy individuals presents challenges primarily because it involves collecting specimens directly from human subjects, which raises both ethical and financial considerations. Therefore, conducting surveillance through samples collected from sewage or toilet waste offers a more practical and sensible approach (Aarestrup and Woolhouse, 2020).

Metagenomics is an effective method for AMR surveillance and has been widely used in AMR studies, including wastewater sampling from airplanes (Petersen et al., 2015; Heß et al., 2019), municipal sewage (Hendriksen et al., 2019; Munk et al., 2022; Becsei et al., 2024), as well as hospital wastewater (Perry et al., 2021; Lepper et al., 2023; Ma et al., 2024). While several studies have performed AMR surveillance in public toilet settings, such as surface swabbing in restrooms (Mkrtchyan et al., 2013) and airport/aircraft lavatory wastewater sampling (Liu et al., 2025), the use of public-toilet waste from general public restrooms to directly monitor local population resistomes remains underexplored.

The aim of this study is to evaluate the feasibility of population-based metagenomic resistome surveillance using sampling at public toilets from different socioeconomic settings (low-, middle-, and high-income) in Bangkok as an example. We then explore the association between income settings and the diversity and abundance of AMR. We find significantly higher abundances in low-income settings compared to middle- and high-income settings, showing both the potential of the sampling strategy and supporting previous findings comparing countries.

## 2 Materials and methods

### 2.1 Sample location and collection

Sampling was conducted in areas representing three distinct income groups—low-, middle-, and high income—within Bangkok, Thailand. For the low-income group, toilet waste samples were collected from fresh markets located near high-density communities, such as government flats and rented housing units with low monthly rental costs, which reflect the characteristics typically observed among populations with limited economic resources (Chaiyaseth, 2024).

Middle-income samples were obtained from markets situated further away from densely populated zones, typically adjacent to condominium complexes with moderate rental prices, reflecting average socioeconomic conditions. High-income samples were collected from luxury residential areas, characterized by high-end properties valued in the multimillion-baht range.

**Figure 1:**
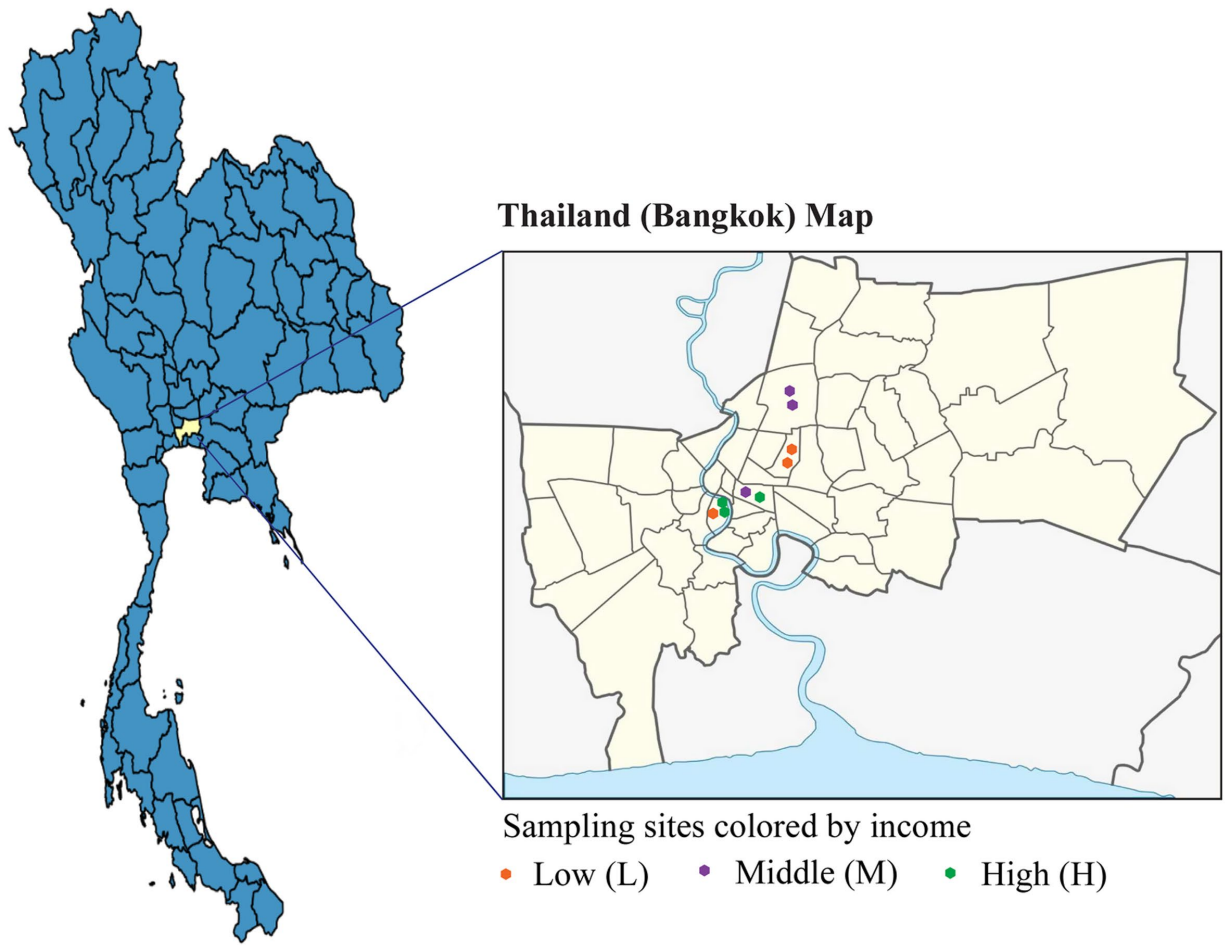
Sampling sites in this study. The sampling sites in Bangkok, Thailand, are classified into three income groups (low, middle, and high income). The colors orange, purple, and green represent the low, middle, and high-income groups, respectively.

Each income group was sampled at three different locations, with sampling conducted in triplicate to ensure the reliability and reproducibility of the data (Klanreungsang and Suppawimut, 2022; Sanit and Gajaseni, 2023; Figure 1).

At each location, 1,000 mg samples of toilet waste (untreated toilet waste) were collected in sterile tubes containing 9 mL of preserving solution (DNA/RNA Shield, Zymo Research). Sampling occurred once per day for three consecutive days at each location between March and April 2023. All samples were properly labeled, stored in a cold container, and transported to the King Mongkut’s University of Technology Thonburi (KMUTT) laboratory in Bangkok for DNA extraction.

### 2.2 DNA extraction and metagenomic sequencing

For each sampling site, the collected triplicate samples were combined into one sample. DNA extraction was carried out using the QIAmp Fast DNA Stool Mini Kit with a modified protocol (Knudsen et al., 2016). The obtained DNA was stored at −20 °C until sequencing. DNA from all samples was mechanically sheared using ultrasonication to a targeted fragment size of 300 bp (Covaris E220 Evolution). The library preparation was performed using the KAPA PCR-Free Library Preparation Kit (Roche), following the manufacturer’s instructions. The 27 libraries were multiplexed and sequenced on the NovaSeq 6,000 platform (Illumina), using 2 × 150 bp paired-end sequencing per flow cell.

### 2.3 Sequencing data processing and mapping

The sequencing reads were trimmed with FoodQCPipeline (CGE, 2016) using default settings. Briefly, reads were evaluated for quality using FastQC v0.11.5 (Bioinformatics, B, 2016), followed by trimming of sequencing adapters using bbduk2 (part of BBtools v36.49)^1^ with an internal database. Subsequently, the trimmed raw reads were re-evaluated for quality using FastQC.

All reads that passed quality control (QC) met the following criteria:

1. read length ≥ 50 base pairs (bp),
2. Phred score per base ≥ 20, and
3. adapters removed.

Trimmed reads were used as input for mapping using KMA v1.4.2 (Clausen et al., 2018) with the following databases:

1. PanRes v1.0.1, a comprehensive collection of resistance genes (Martiny et al., 2023) comprising several public databases, as well as additional databases for metal and biocide resistance; and
2. genomic v2022-05-24, a database for bacterial identification based on NCBI whole genome sequencing (WGS; in-house CGE database).
  There is considerable overlap between the different antibiotic resistance gene (ARG) databases; however, in this study, we focus on ResFinder, which contains ARGs that have been mobilized from their original context and are therefore considered acquired; ResFinderFG and ResFinderNG (also called CsabaPal), which are databases of ARGs identified through functional cloning, and databases for metal and biocide resistance.

### 2.4 Total resistance abundance analysis

The total resistance abundance of each sample was quantified in Fragments Per Kilobase per Million mapped reads (FPKM) units (Hendriksen et al., 2019; Munk et al., 2022), in which the fragments of a specific gene were normalized by the base-pair length of the reference sequence and the total number of mapped fragments. Total resistance abundance per sample was calculated as a relative abundance value (0–1).

The FPKM-assigned sequencing fragments were normalized using ARG_Length_ and Bacteria_Depth_ according to the following equation:


$$\;\text{Total resistance abundance}=\frac{\mathrm{AR}G_{\text{Fragments}}}{\mathrm{AR}G_{\text{Length}}\times \text{Bacteri}a_{\text{Depth}}}\times 10^{9}$$

where ARG_Fragments_ is the number of sequencing fragments assigned to the reference sequence, ARG_Length_ is the length of the ARG reference sequence, and Bacteria_Depth_ is the total number of reads mapped to all bacterial genomes in the NCBI WGS database.

### 2.5 Differential analysis of the resistome and bacterial community

For this analysis, we used the read counts from mapping against the AMR and genomic databases. Differences in abundance were calculated using ALDEx2 v1.32.0 R package (Fernandes et al., 2013; Fernandes et al., 2014; Gloor et al., 2016; Nixon et al., 2022).

To account for the compositional nature of the data, the abundance input (size-adjusted read counts) was transformed to CLR (centered log-ratio) values to determine abundance differences using Welch’s test, followed by a Benjamini–Hochberg (BH) false-discovery rate (FDR) correction (Benjamini and Hochberg, 1995).

The variation within groups and between groups was visualized in effect plots, highlighting statistically significant differences with FDR < 0.05 in red.

### 2.6 Biodiversity measures

To compare differences in ARGs and bacterial genera among income groups, we estimated richness (Chao1 richness) and diversity (Shannon and Simpson) indices. The estimation was calculated in R using the vegan package (Oksanen et al., 2022).

### 2.7 Data visualization

Sampling sites in Bangkok were visualized with MapChart.^2^ Several R packages were used for visualization. Principal component analysis (PCA) was visualized using factoextra (Kassambara and Mundt, 2020). Relative abundance bar charts were generated using ggplot2 (Wickham, 2016). Dispersion and effect plots were visualized using AlDEx2 (Fernandes et al., 2013; Fernandes et al., 2014; Gloor et al., 2016; Nixon et al., 2022). Diversity measures were visualized using vegan (Oksanen et al., 2022). Heatmaps were generated using pheatmap (Kolde, 2019).

## 3 Results

### 3.1 Summary of sequencing quality

The average number of QC-passed bases was 9,873 ± 1,365 megabases (Mb). The average number of QC-passed reads was 70 ± 8 × 10^6^ reads. The proportion of qualified reads that could be mapped to the bacterial genome (genomic database) was 22.9 ± 15.2%. Neither qualified bases nor reads were significantly different among the income groups (*p* > 0.05; Supplementary Figure 1).

### 3.2 Differences in total resistance abundance between income groups

We determined the resistance abundance of genes (resistome) conferring resistance to antibiotics, metals, and biocides.

**Figure 2:**
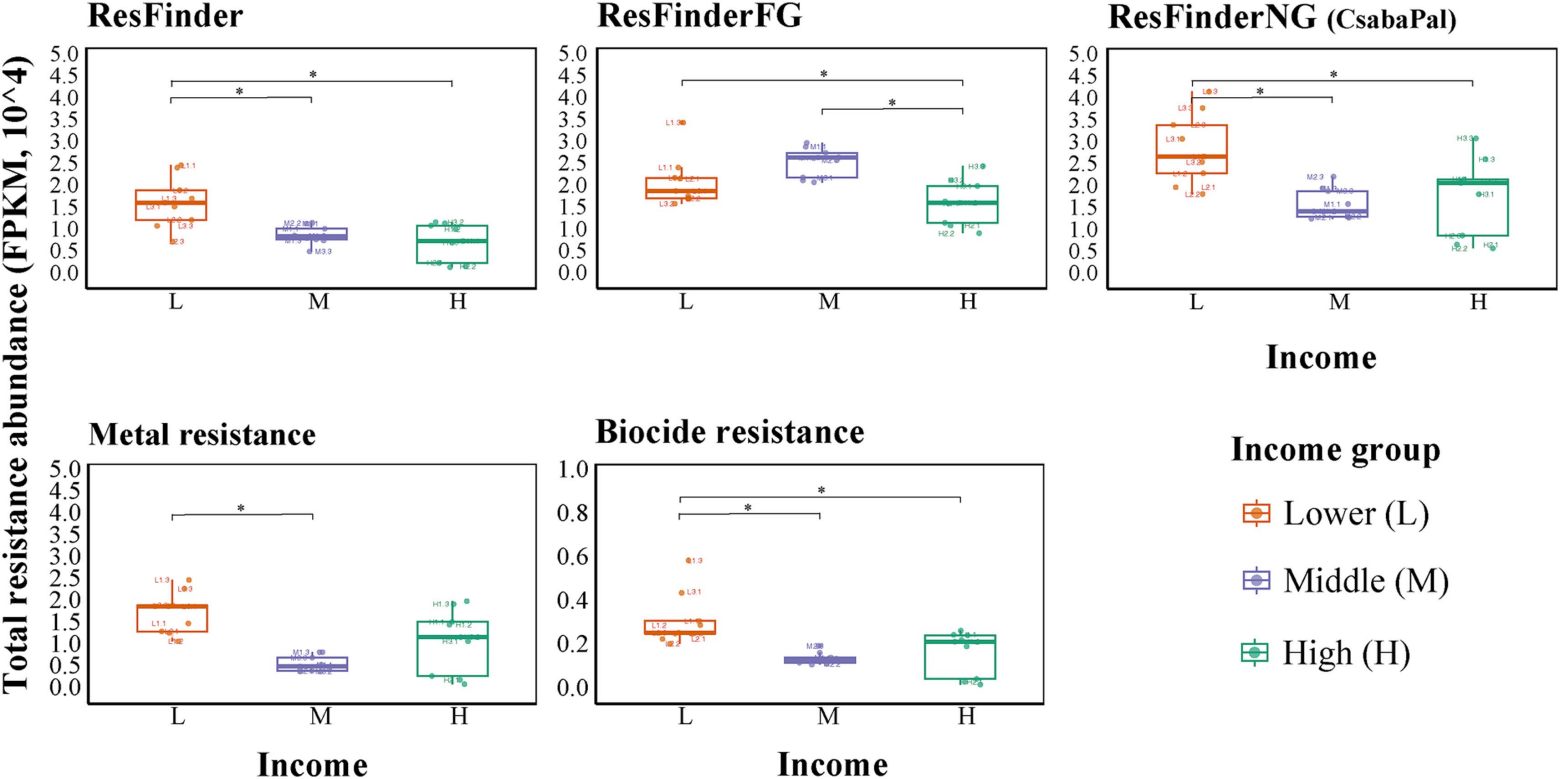
Trend of resistance abundance in different databases. The differences of total resistance abundance (fragments per kilobase per million mapped reads (FPKM)) among income groups were tested. Differences are analyzed using Tukey’s honestly significant difference test with bias-corrected and accelerated intervals (95%) when normality and homogeneity assumptions were met, or pairwise Wilcoxon rank-sum tests when normality or homogeneity assumptions were not met (*p* < 0.05).

A clear trend was observed in which total resistance abundance (FPKM) was significantly higher in low-income areas compared to middle- and high-income areas (*p* < 0.05). For instance, based on the ResFinder database, the total resistance abundance (FPKM) for the low-, middle-, and high-income groups was 1.73 ± 0.58, 0.97 ± 0.21, and 0.81 ± 0.43 (×10^4^), respectively, indicating a statistically significant difference (Figure 2).

Similar significant patterns of total resistance abundance were also observed in both the ResFinderNG and biocide databases. In the case of metal resistance genes (MRGs), the low-income group also showed higher abundance; however, this was statistically significant only when compared to the middle-income group. For the ResFinderFG database, both low- and middle-income settings exhibited higher resistance abundances than the high-income setting (Figure 2).

### 3.3 Differences in resistome diversity among income groups

#### 3.3.1 Differences within antimicrobial class

**Figure 3:**
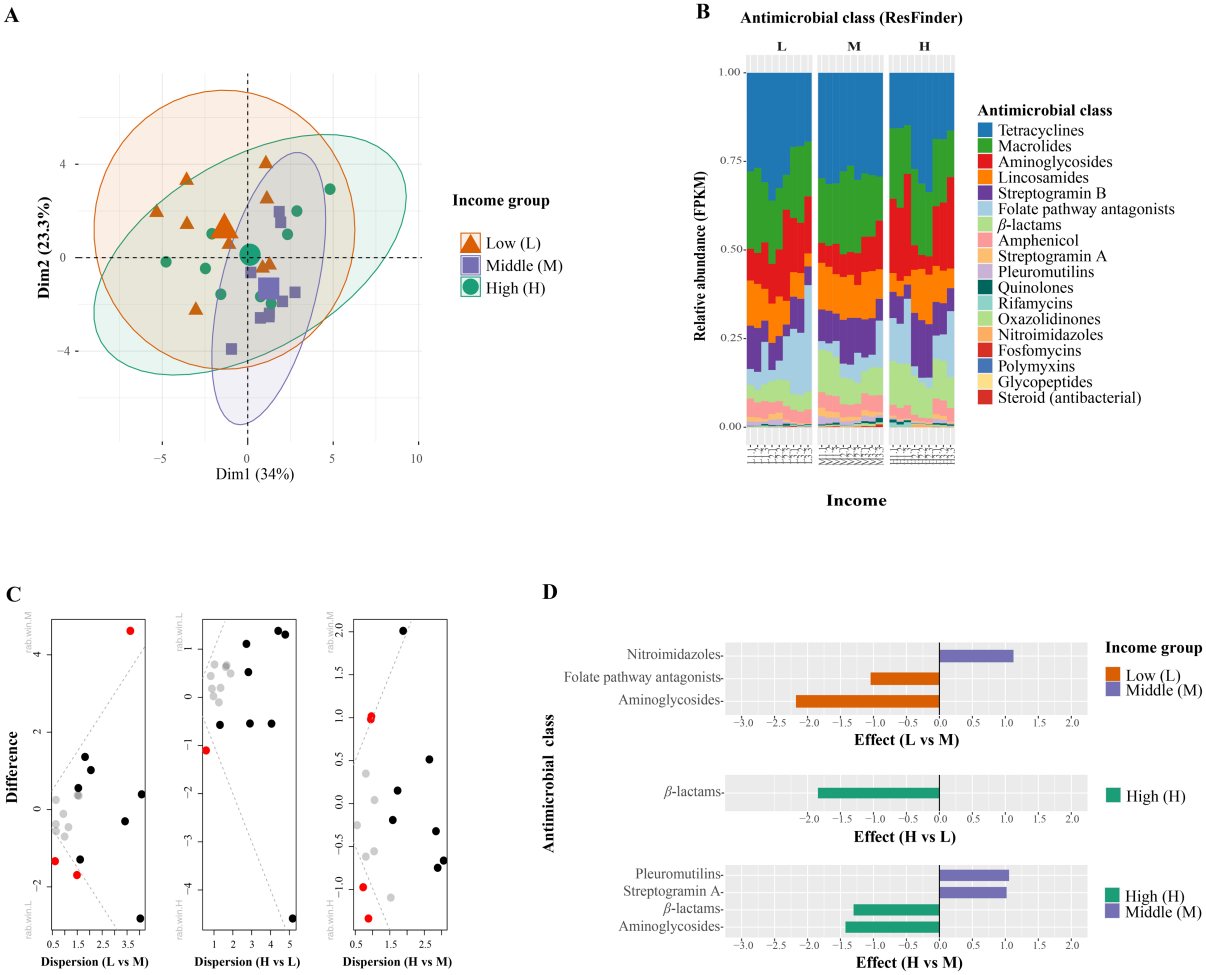
Differences in antimicrobial classes among three different income groups. **(A)** Principal component analysis based on centered log-ratio transformation across three income groups [low (L), middle (M), and high (H)]. **(B)** Relative abundance of antimicrobial classes. **(C)** Dispersion shows differences in antimicrobial classes between two income groups: (1) low vs. middle, (2) high vs. low, and (3) high vs. middle. Red dots indicate statistically significant different (*p* < 0.05) based of Welch’s test with Benjamini–Hochberg correction. **(D)** Effect plot shows the effect size and significantly different antimicrobial classes. The colors orange, purple, and green represent the low-, middle-, and high-income groups, respectively.

Based on the ResFinder antimicrobial class categorization, the most dominant antimicrobial classes detected were tetracyclines, macrolides, and aminoglycosides (Figure 3).

We observed several differences in antimicrobial class abundance across income groups. Notably, *β*-lactam resistance genes showed significantly higher abundance in high-income areas compared to low- and middle-income areas (*p* < 0.05; Figure 3). Additionally, low-income settings exhibited higher abundances of genes conferring resistance to aminoglycosides and folate pathway antagonists compared to other income groups (Figure 3).

#### 3.3.2 Differences within antimicrobial resistance genes

When analyzing AMR gene abundance based on the ResFinder database, we observed that sul2 and bla_OXA-347_ showed the highest proportions of resistance abundance across all three income groups.

**Figure 4:**
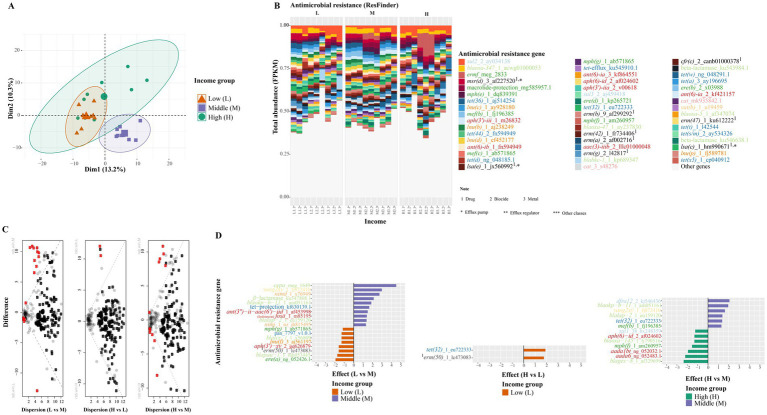
Differences in antimicrobial resistance gene (ResFinder) among three different income groups. **(A)** Principal component analysis based on centered log-ratio transformation across three income groups [low (L), middle (M), and high (H)]. **(B)** Relative abundance of antibiotic resistance genes (ARGs). **(C)** Dispersion shows differences in ARGs between two income groups: (1) Low vs. middle, (2) High vs. low, and (3) High vs. middle. Red dots indicate statistically significantly differences (*p* < 0.05) based on Welch’s test with Benjamini–Hochberg correction. **(D)** Effect plot shows the effect size and significantly different ARGs. The colors orange, purple, and green represent the low-, middle-, and high-income groups, respectively.

Upon comparing resistance abundance by gene, we found that certain bla genes, bla_GES-8-1_ and bla_OXA-145_, were more abundant in high-income settings compared to middle-income settings. In contrast, other *β*-lactam-associated genes, including cepa, bla_OKP_, and bla_LAP_, were more abundant in middle-income settings compared to low-income settings (Figure 4).

Although we previously reported significant differences in overall *β*-lactam class abundance between low- and high-income groups, we did not observe significant differences in specific β-lactam genes between these two settings (Figure 4; Supplementary Figure 2).

Based on AMR genes mapped to the ResFinderFG dataset, we observed a higher relative abundance of sul2 (light blue), tetracycline resistance genes (tet; blue), *β*-lactam-related genes (light green), and van-ligase glycopeptide resistance genes (yellow). When comparing gene abundance between groups, starting with the low- and middle-income settings, we found that functional β-lactamase-related genes and tet genes were more abundant in the low-income group compared to the middle-income group. In contrast, the middle-income group showed a higher abundance of van-ligase genes than the low-income group.

Notably, van-ligase glycopeptide-related genes were observed in higher proportions in both middle- and high-income settings compared to the low-income setting. Therefore, functional β-lactamases were more abundant in low-income settings, whereas van-ligase genes were more prominent in middle- and high-income settings (Supplementary Figure 3).

Based on the ResFinderNG database, which contains a curated dataset of novel resistance genes, we observed notable resistance gene profiles across different income settings. Overall, a high relative abundance of genes related to polymyxin and β-lactam resistance was detected. In particular, β-lactam-related genes (light green) were more abundant in the low-income setting compared to the middle-income setting. Additionally, polymyxin-related genes (blue) were predominantly observed in both low- and middle-income settings (Supplementary Figure 4).

#### 3.3.3 Metal resistance

When analyzing resistance genes from the metal resistance datasets, we identified a wide range of genes conferring resistance to arsenic, mercury, tellurium, copper, iron, lead, nickel, chromium, and zinc, with varying levels of relative abundance across the income groups.

These resistance mechanisms were mediated primarily by metal resistance proteins. We observed significant differences in MRG abundance between the low- and middle-income groups. Notably, in the low-income group, we found a higher abundance of genes encoding resistance to iron (acn), mercury (merE, merA, merD, merT), and copper (dnaK, copA) compared to the middle-income group (Supplementary Figure 5).

#### 3.3.4 Biocide resistance

When focusing on the biocide resistance datasets, we observed a high relative abundance of sobd and tric, which confer resistance to peroxides and phenols, respectively. Both genes were found to be more abundant in low- and high-income settings compared to the middle-income settings (Supplementary Figure 6).

### 3.4 Differences in bacterial communities among income groups

We identified more than 2,432 genera across 49 phyla. Among these genera, the top five by relative abundance were *Bacteroides* spp., *Phocaeicola* spp., *Faecalibacterium* spp., *Advenella* spp., and *Prevotella* spp.

When comparing genera across income groups, variations were observed both in genus composition and relative abundance. However, *Sediminibacterium* spp. from the high-income areas showed higher abundance compared with the low- and middle-income areas (Supplementary Figure 7).

### 3.5 Alpha diversity

We measured alpha diversity indices (Chao1 richness, Shannon, and Simpson) across different income groups using all three databases.

For ARG abundance, the Chao1 richness, Shannon, and Simpson indices tended to be higher in the middle- and/or high-income groups compared with the low-income group (*p* < 0.05). However, no statistically significant differences in the alpha diversity of bacterial genera were observed among the income groups (Supplementary Figure 8).

## 4 Discussion

This study collected toilet waste from three different income areas to investigate the resistome using Bangkok as a model for surveillance. This is the first study to use metagenomics (including ARGs and metal and biocide resistance) from public toilets from three different income groups in Thailand as a pilot study for public health surveillance and future AMR monitoring. Previous studies have utilized sewage collected at public restrooms (Mkrtchyan et al., 2013), sewage treatment plants (Hendriksen et al., 2019; Munk et al., 2022; Jespersen et al., 2023), hospitals (Perry et al., 2021), and long-distance airplanes (Petersen et al., 2015; Liu et al., 2025), while we show here the potential of using public toilet waste and linking this to potential socioeconomic groups.

We found higher abundances of acquired ARGs (based on the ResFinder database) in low-income areas compared to middle- and high-income areas. This is in agreement with previous studies using the same database comparing data from different countries that also show the importance of socioeconomic parameters in shaping the abundance of AMR (Hendriksen et al., 2019; Nadimpalli et al., 2020; Njage et al., 2023). Income is thus likely a significant factor in the development of AMR, and in terms of AMR surveillance, low-income communities should be strategic areas for governments aiming to prevent infections and control the spread of AMR.

These areas might face limitations in maintaining infrastructure. Regular checks and maintenance of these systems are crucial to prevent the spread of waste to humans and the environment. Higher-income areas typically have robust systems to treat and contain waste locally before its release, helping to reduce the risk of AMR development. Thus, enhancing sanitation, healthcare, and education—as outlined in the Sustainable Development Goals—could be effective strategies for reducing the global burden of AMR (Anonymous, 2019). This also aligns with the World Health Organization’s water, sanitation, and hygiene concept, which underscores the importance of water, sanitation, and hygiene in combating AMR development, especially in low- and middle-income communities or densely populated areas. However, epidemiological data besides socioeconomic status were not available for this study, and several factors could influence these results, including waste management practices, environmental conditions, and personal AMU.

Most published studies on AMR in wastewater have focused on hospital effluents or wastewater treatment plants and consistently report high relative abundances of ARGs associated with aminoglycosides, *β*-lactams, macrolide–lincosamide–streptogramin, and tetracyclines (Knight et al., 2024; Silvester et al., 2025a; Silvester et al., 2025b). Metagenomic studies also reveal regional differences in AMR abundance and diversity between Europe, North America, and Oceania vs. Africa, Asia, and South America (Smith et al., 2024). In South Africa, wastewater treatment works serving different income communities showed differences in AMR class distribution, with macrolides and macrolide–streptogramin B resistance predominating in urban high-income areas and aminoglycoside resistance in semi-urban low-income areas (Hendriksen et al., 2019). A study examining toilet waste from international airplanes reported higher abundance and diversity of ARGs, including bla_CTX-M_, in flights from South Asia compared with North America (Petersen et al., 2015). However, due to differences in study design and methodology, a direct comparison of AMR percentages across studies remains challenging.

We observed that ARGs conferring resistance to *β*-lactams, aminoglycosides, and tetracyclines differed across income-level areas, with β-lactam resistance genes showing particularly distinct patterns in particular income groups. Our metagenomic analysis revealed the widespread presence of β-lactam resistance genes in toilet waste; however, a limitation of this approach is that the bacterial host cells carrying the resistance genes remain unidentified unless metagenomic-assembled genomes are investigated.

In addition to the ResFinder database of acquired ARGs, we also analyzed ARGs identified through functional cloning, both for currently used antibiotics (ResFinderFG database) and for novel antibiotics under development (ResFinderNG). Notably, ARGs associated with the functional groups of β-lactamases and tetracycline protection proteins differed between income groups. For functional groups related to vancomycin resistance, particularly ligase activity (van-ligase), these genes were more prominent in middle- and high-income areas.

For the ARG analysis using the ResFinderNG database, we found two important insights. First, we still observed the persistence of ARGs related to β-lactams. Second, we observed the presence of novel ARGs associated with polymyxin. The detection of polymyxin-related resistance genes is of particular concern, as it may indicate emerging threats to this antibiotic class. Furthermore, ResFinderNG identified a substantial number of putative or uncharacterized resistance genes, many of which are currently unknown in terms of function and clinical significance. These findings highlight the limitations of existing databases and point to the urgent need for further functional characterization and validation of these novel genes in future studies.

Regarding resistance to metal and biocides, we observed resistance genes associated with several potentially hazardous agents. Among these, arsenic resistance genes exhibited the highest relative abundance, followed by those related to mercury and copper, which also showed dominant prevalence across samples. Studies have reported higher levels of metal contamination and the use of metal-based disinfectants in low- and middle-income settings, particularly in low-income settings (Amin et al., 2024; Hounmanou et al., 2025). These conditions are often associated with inadequate waste management and industrial discharge, leading to environmental accumulation of heavy metals. Notably, previous research has documented a high prevalence of MRGs, suggesting strong selective pressure from prolonged metal exposure (Gendy et al., 2020; Murray et al., 2024). Biocides are chemical substances or microorganisms used for a variety of purposes and applications. Bacteria often develop resistance to these agents through various mechanisms, including reducing biocide penetration, activating efflux pumps, and enzymatic degradation (Maillard, 2018).

Because public toilets—particularly those in markets and other public venues—are used by people from both local communities and transient populations, the resistome profiles detected may capture AMR from a wider geographic catchment than the immediate surroundings. This mobility factor could make such sites valuable for tracking AMR dissemination patterns and identifying emerging threats that cross community or regional boundaries in future studies.

Our bacterial community analysis revealed a high relative abundance of genera typically associated with the human gut microbiome, such as *Bacteroides*, *Phocaeicola*, *Faecalibacterium*, and *Prevotella* (Bresser et al., 2022; Hou et al., 2022), consistent with fecal origin and supporting the validity of public toilet waste as a proxy for human-derived microbiota that also carry ARGs. Public toilets, especially in high-traffic areas, may therefore serve as nodes for the persistence and possible dissemination of MDR bacteria.

In conclusion, we highlighted significant differences in resistome abundance in lower-income groups compared to middle- and high-income groups. Our findings showed clear differences in antimicrobial classes and ARGs among income groups. *β*-lactam and tetracycline resistance genes showed different abundance patterns in high-income groups compared with low- and middle-income groups. This study provides the first metagenomic analysis of toilet waste across income-level settings. It can serve as a valuable model for establishing appropriate surveillance mechanisms for AMR in Thailand and potentially in other regions.

## Data Availability

The datasets presented in this study can be found in online repositories. The names of the repository/repositories and accession number(s) can be found in the article/Supplementary material.

## Footnotes

1
https://jgi.doe.gov/data-and-tools/software-tools/bbtools/

2
https://www.mapchart.net/
