# Supplementary material for: Comparative resistome from toilet waste in three different income areas, Bangkok, Thailand
Source: Front Microbiol. 2026 Mar 25;17:1790551. doi: 10.3389/fmicb.2026.1790551 (PMC13057367; doi:10.3389/fmicb.2026.1790551)
Supplement: Supplementary file 1 [file Table_1.DOCX]

Supplementary Material

# Supplementary Data

The supplementary (excel file) includes multiple sheets containing (sheet01) sample metadata, (sheet02) sequencing quality control (QC), (sheet03) differences in QC parameters (quality bases and quality reads) between income groups, (sheet04) differences in FPKM across databases between income groups, and (sheet05) differences in diversity indices on genus and databases.

# Supplementary Figures

**Supplementary Figure 1.** Total qualified bases and reads in this study. The qualified bases (MB-million bases) and reads beyond the sequencing quality checking were classified into low (L), middle (M), and high (H) income groups. The differences between income groups of the qualified bases and reads were tested with Pairwise-Wilcoxon Rank Sum Tests with *p*-value < 0.05.

**Supplementary Figure 2.** Clustering of antimicrobial resistance genes in three different income groups based on ResFinder database. The heatmap displays the clustering of the resistome (as AMR genes) featuring the top 50 genes. Size-adjusted read counts of variables across samples were aggregated, filtered for variable value > 0, and used as input data. A Bray-Curtis distance matrix was calculated to assess the similarity in gene abundance profiles. The first 50 high abundance of AMR genes were log-transformed and color-coded from low (black) to high (yellow). The orange, purple and green represent the low-, middle- and high-income groups, respectively.

**Supplementary Figure 3.** Differences of antimicrobial resistance genes (ResFinderFG) in three different income groups. (A) The PCA is measured in three income groups (low (L), middle (M), high (H)). (B) Relative abundance of resistance genes. (C) Dispersion shows the differences of AMR genes between two-income groups; 1) Low vs Middle, 2) High vs Low and 3) High vs Middle. The red dots are significantly different (*p* <0.05) of Welch test with BH correction. (D) Effect shows the effect size and significant AMR genes. The orange, purple and green represent the low-, middle- and high-income groups, respectively.

**Supplementary Figure 4.** Differences of antimicrobial resistance genes (ResFinderNG) in three different income groups. (A) The PCA is measured in three income groups (low (L), middle (M), high (H)). (B) Relative abundance of resistance genes. (C) Dispersion shows the differences of AMR genes between two-income groups; 1) Low vs Middle, 2) High vs Low and 3) High vs Middle. The red dots are significantly different (*p* <0.05) of Welch test with BH correction. (D) Effect shows the effect size and significant AMR genes. The orange, purple and green represent the low-, middle- and high-income groups, respectively.

**Supplementary Figure 5.** Differences of metal resistance genes in three different income groups. (A) The PCA is measured in three income groups (low (L), middle (M), high (H)). (B) Relative abundance of metal resistance genes. (C) Dispersion shows the differences of metal resistance genes between two-income groups; 1) Low vs Middle, 2) High vs Low and 3) High vs Middle. The red dots are significantly different (*p* <0.05) of Welch test with BH correction. (D) Effect shows the effect size and significant metal resistance genes. The orange, purple and green represent the low-, middle- and high-income groups, respectively.

**Supplementary Figure 6.** Differences of biocide resistance genes in three different income groups. (A) The PCA is measured in three income groups (low (L), middle (M), high (H)). (B) Relative abundance of biocide resistance genes. (C) Dispersion shows the differences of biocide resistance genes between two-income groups; 1) Low vs Middle, 2) High vs Low and 3) High vs Middle. The red dots are significantly different (p <0.05) of Welch test with BH correction. (D) Effect shows he effect size and significant biocide resistance genes. The orange, purple and green represent the low-, middle- and high-income groups, respectively.

**Supplementary Figure 7.** Differences of genus in three different income groups. (A) The PCA is measured in three income groups (low (L), middle (M), high (H)). (B) Relative abundance of genus. (C) Dispersion shows the differences of genus between two income groups; 1) Low vs Middle, 2) High vs Low and 3) High vs Middle. The red dots are significantly different (*p* <0.05) of Welch test with BH correction. (D) Effect shows the effect size and significant bacterial genus. The orange, purple and green represent the low-, middle- and high-income groups, respectively.

**Supplementary Figure 8.** The alpha diversity of antibiotic resistance genes and bacterial genus.
The diversity was measured in three different income groups, lower (L), middle (M) and high (H). The diversity indices were measured with Chao1, Shannon and Simpson. The differences between income group of diversity measurement were tested with Tukey’s HSD test + bias-corrected and accelerated interval (95%) (normality + homogeneity) or Pairwise-Wilcoxon Rank Sum Tests (non-normality or homogeneity) with *p*-value < 0.05.
