# Supplementary figures and images for: Comparative resistome from toilet waste in three different income areas, Bangkok, Thailand
Source: Front Microbiol. 2026 Mar 25;17:1790551. doi: 10.3389/fmicb.2026.1790551 (PMC13057367; doi:10.3389/fmicb.2026.1790551)

# Total qualified bases

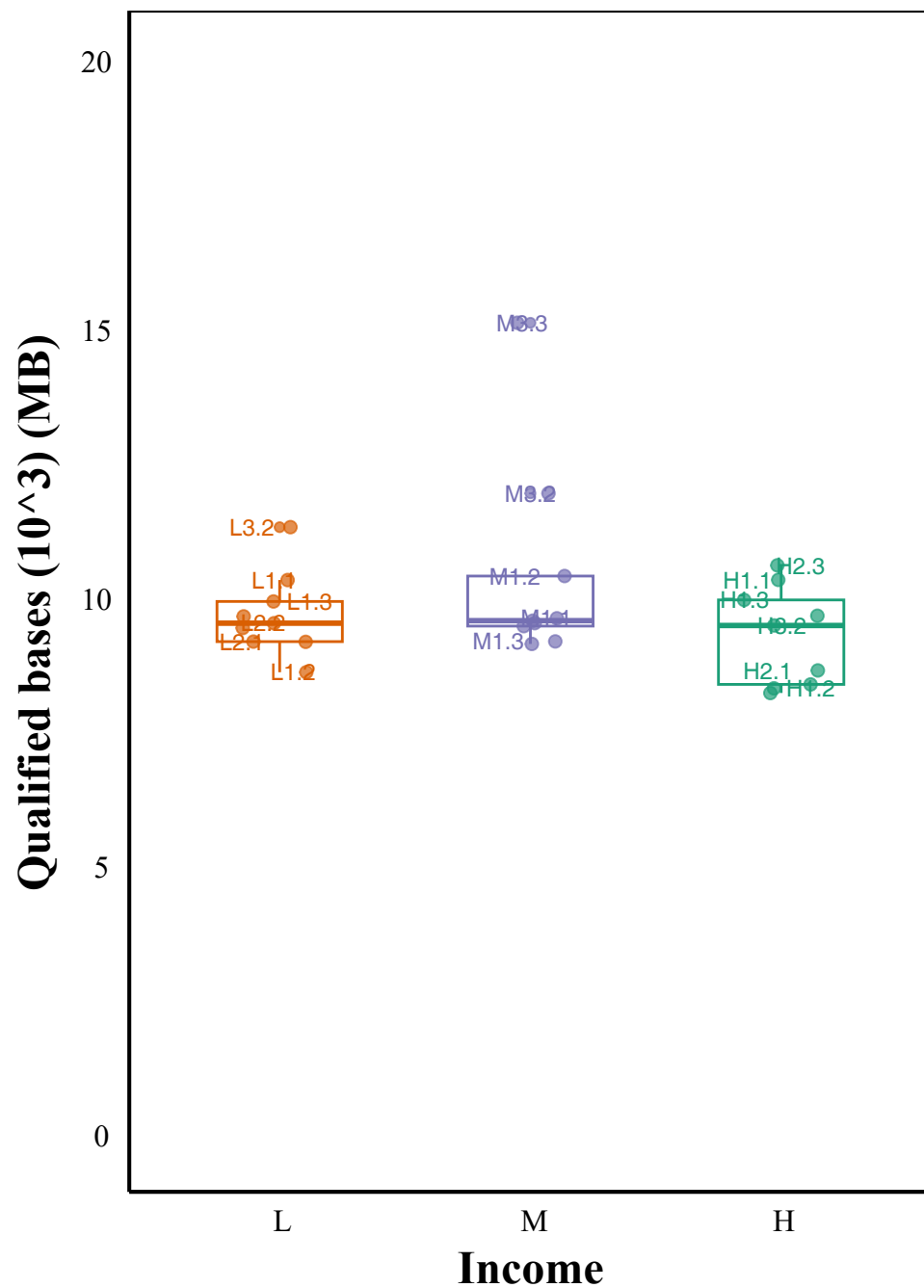

# Total qualified reads

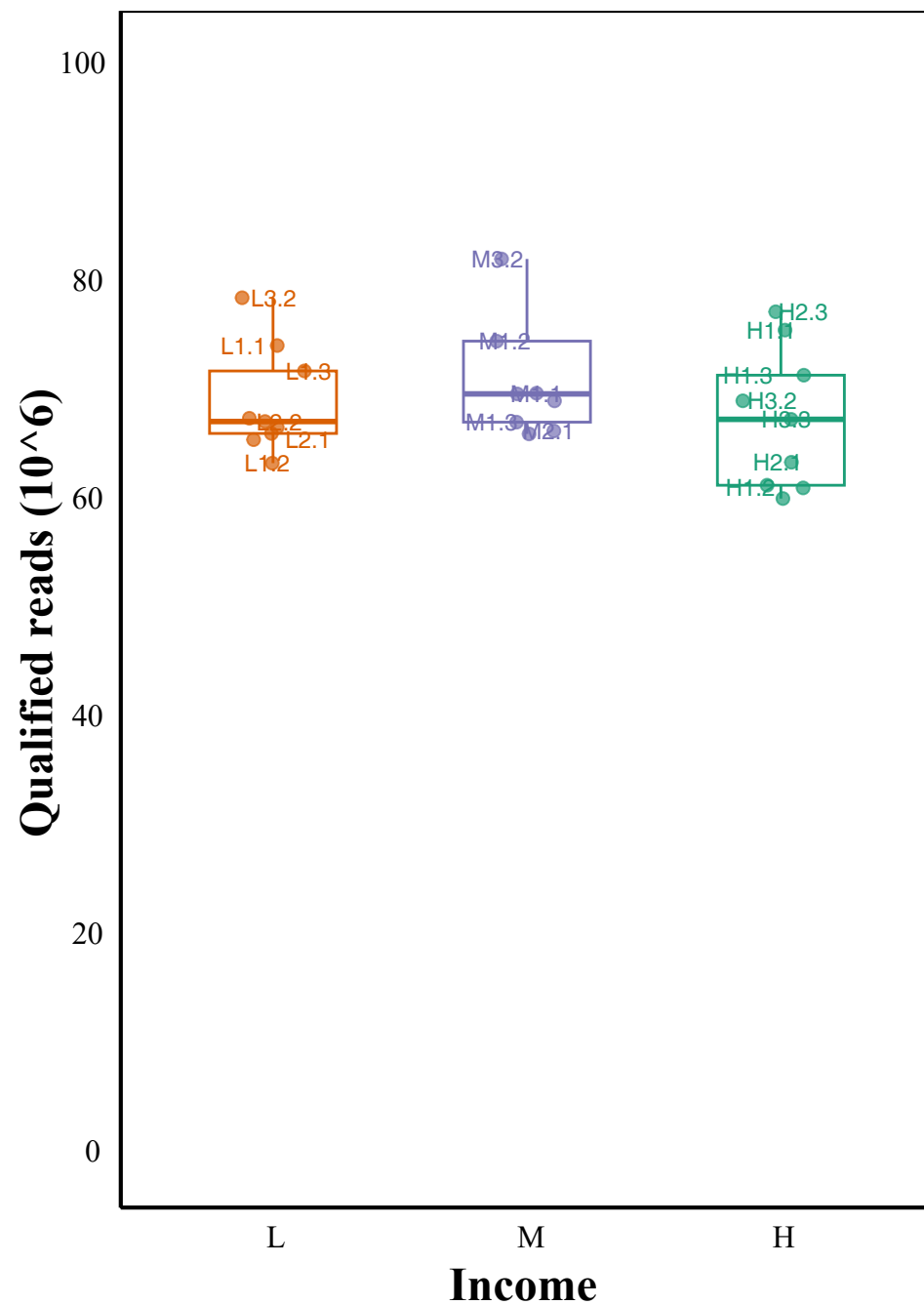

Income group

Lower (L)

Middle (M)

High (H)

Supplement: Supplementary file 3 [file Data_Sheet_1.PDF]

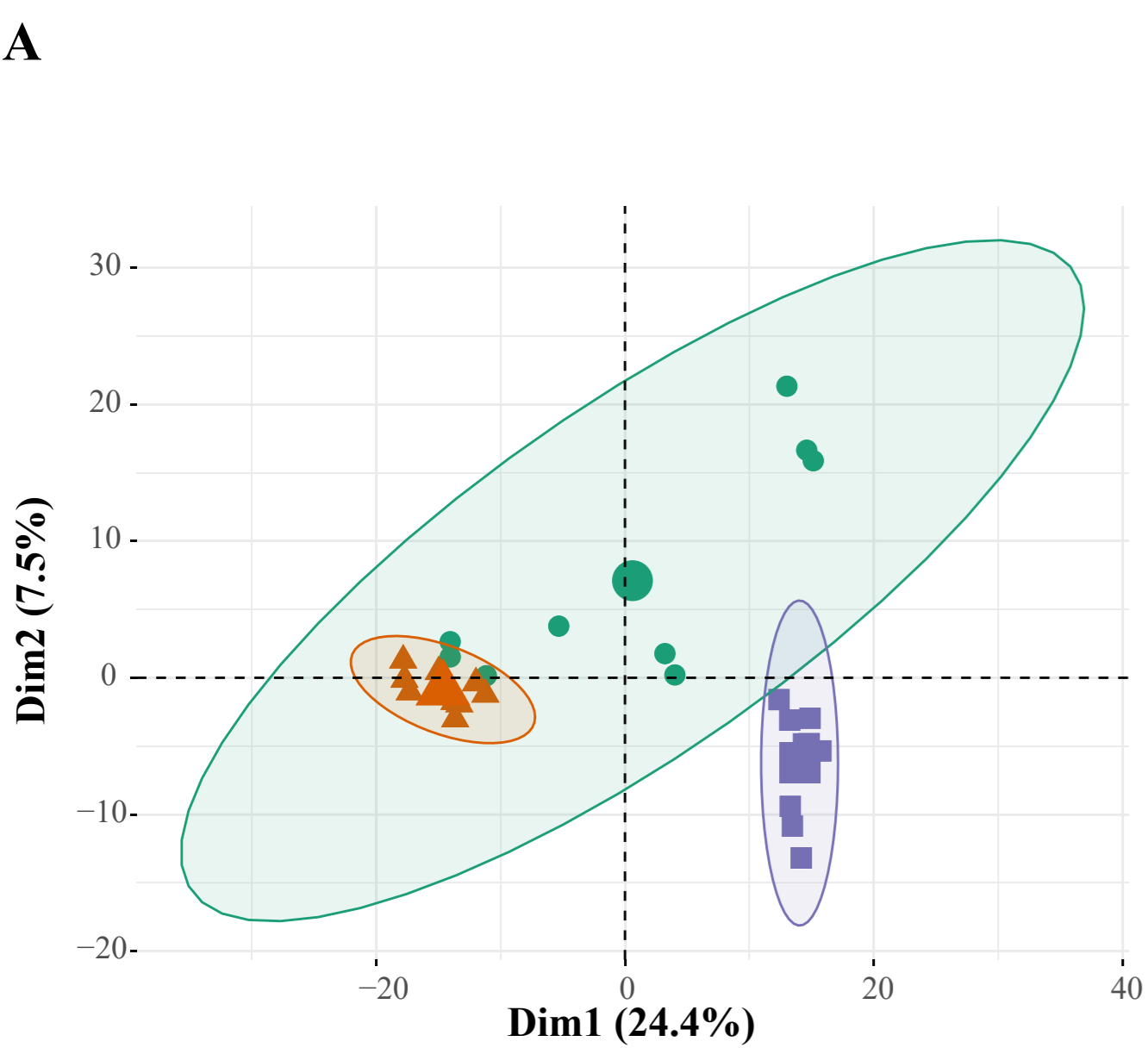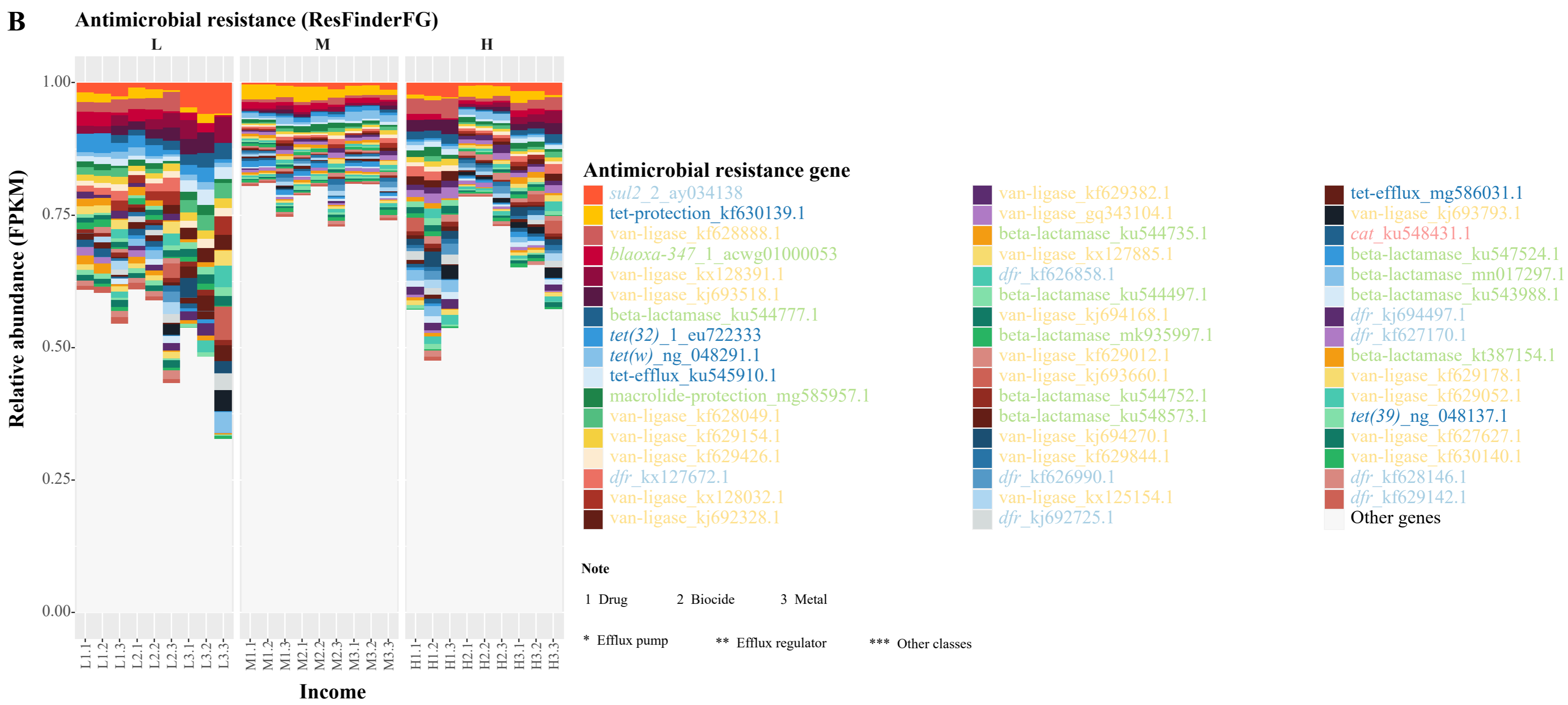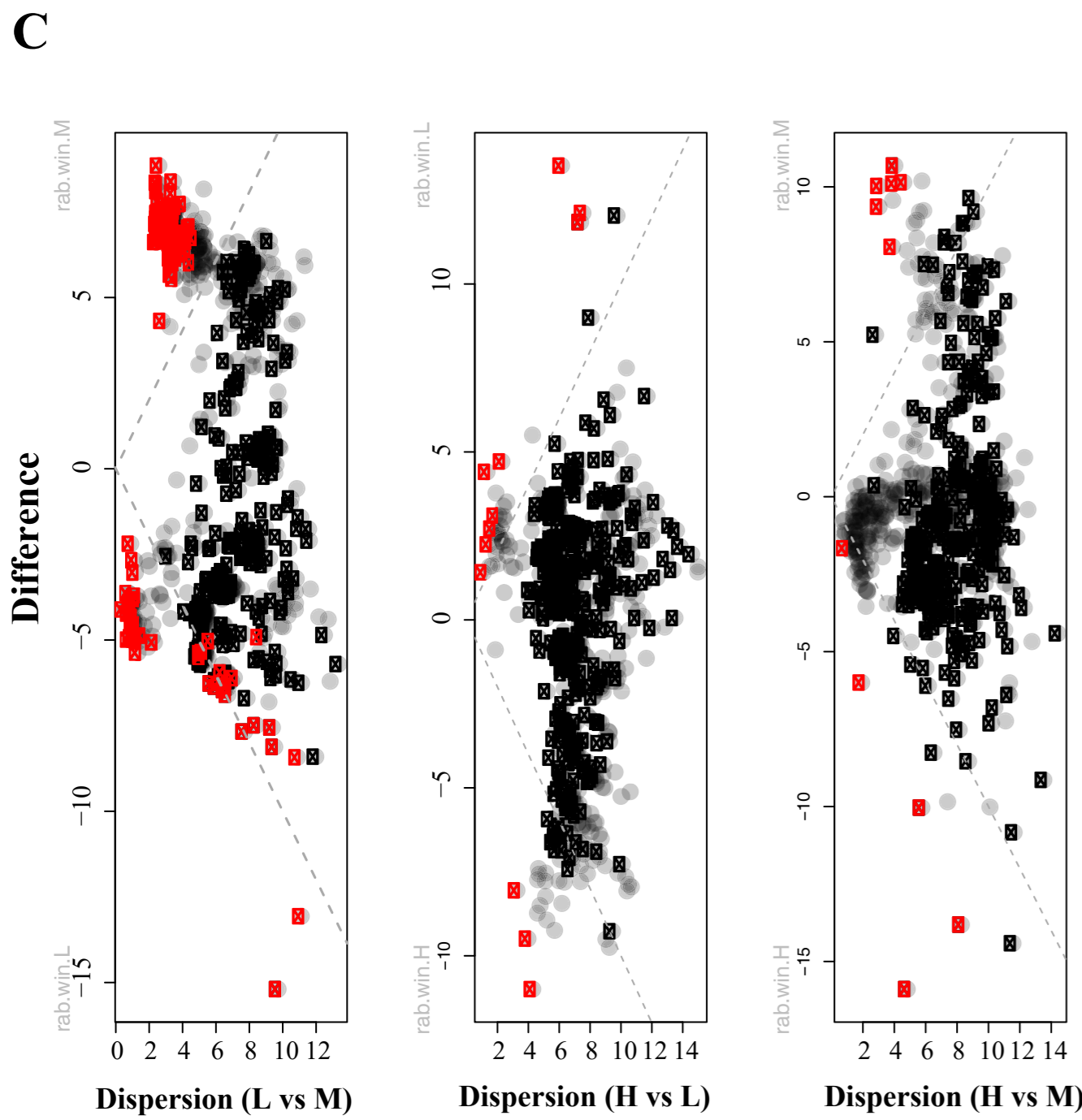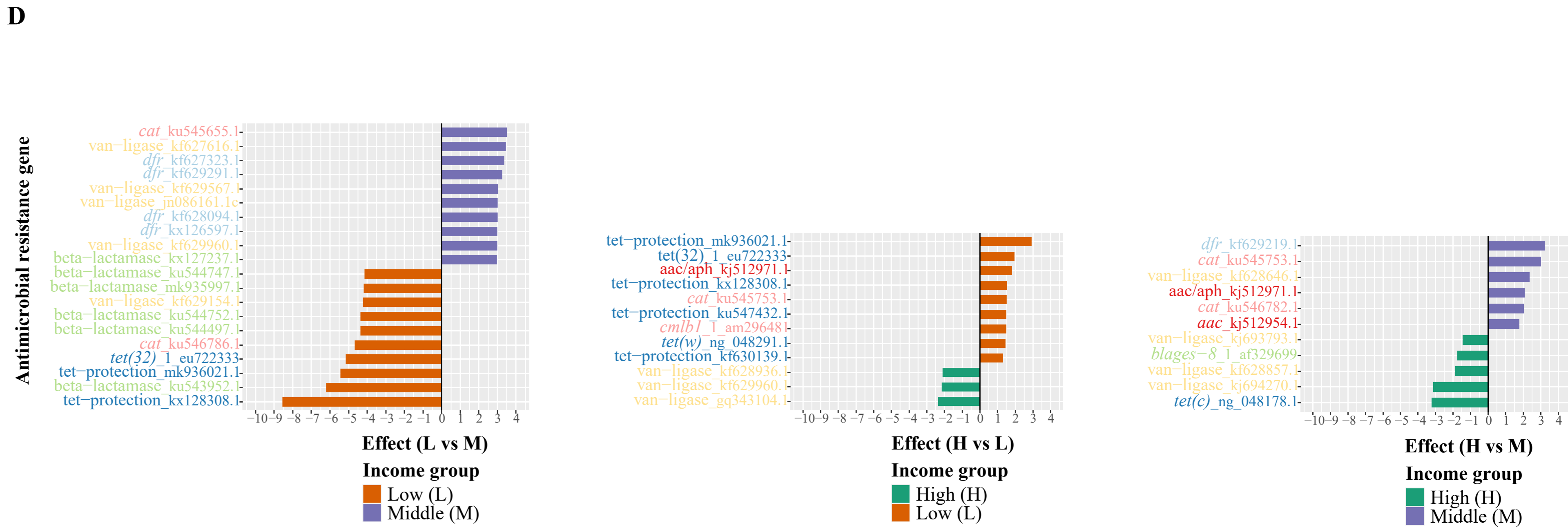

Supplement: Supplementary file 5 [file Data_Sheet_3.PDF]

A

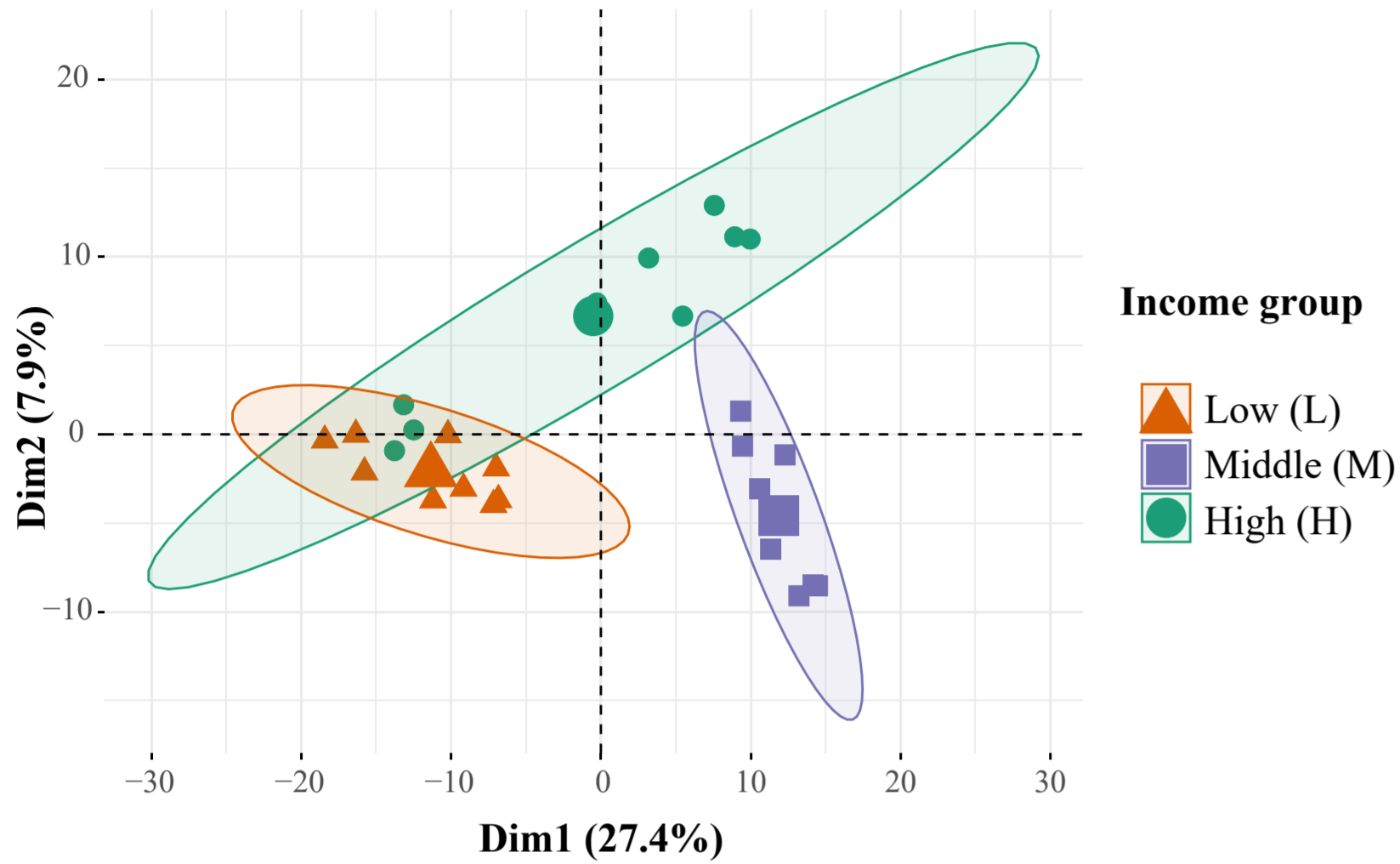

B

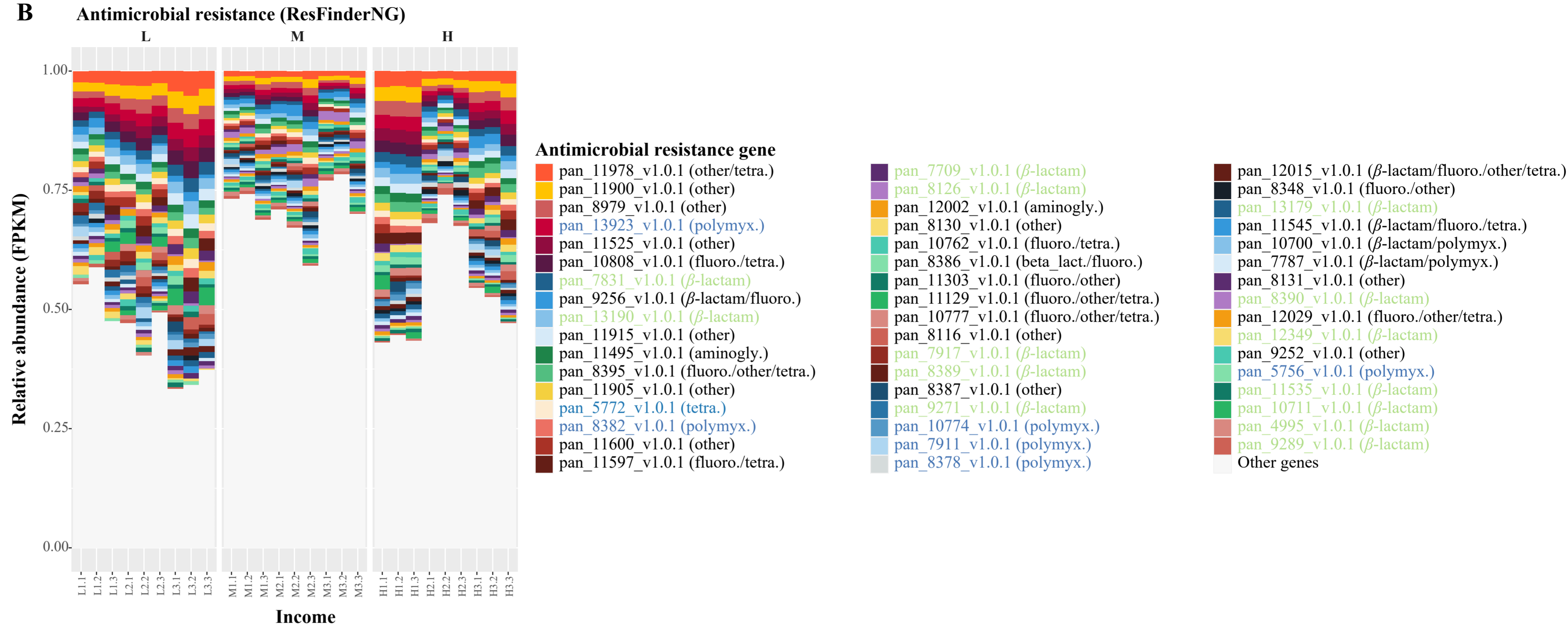

C

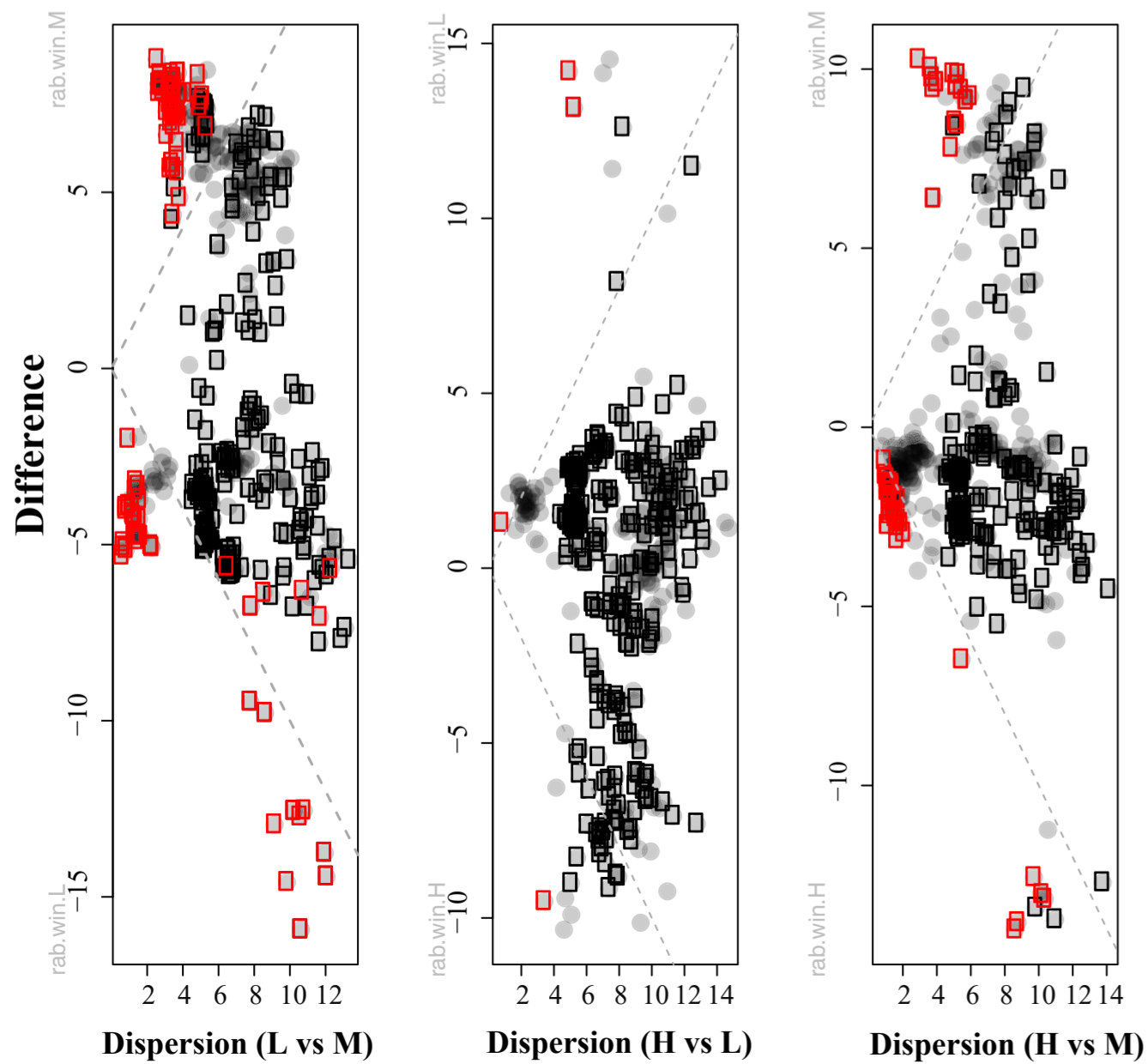

D

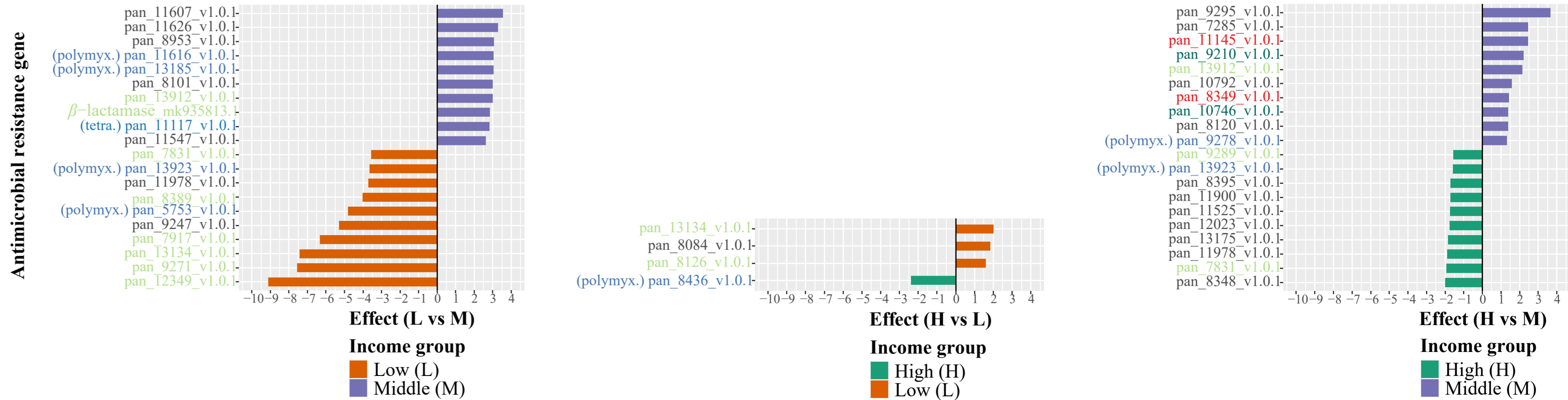

Supplement: Supplementary file 6 [file Data_Sheet_4.PDF]

A

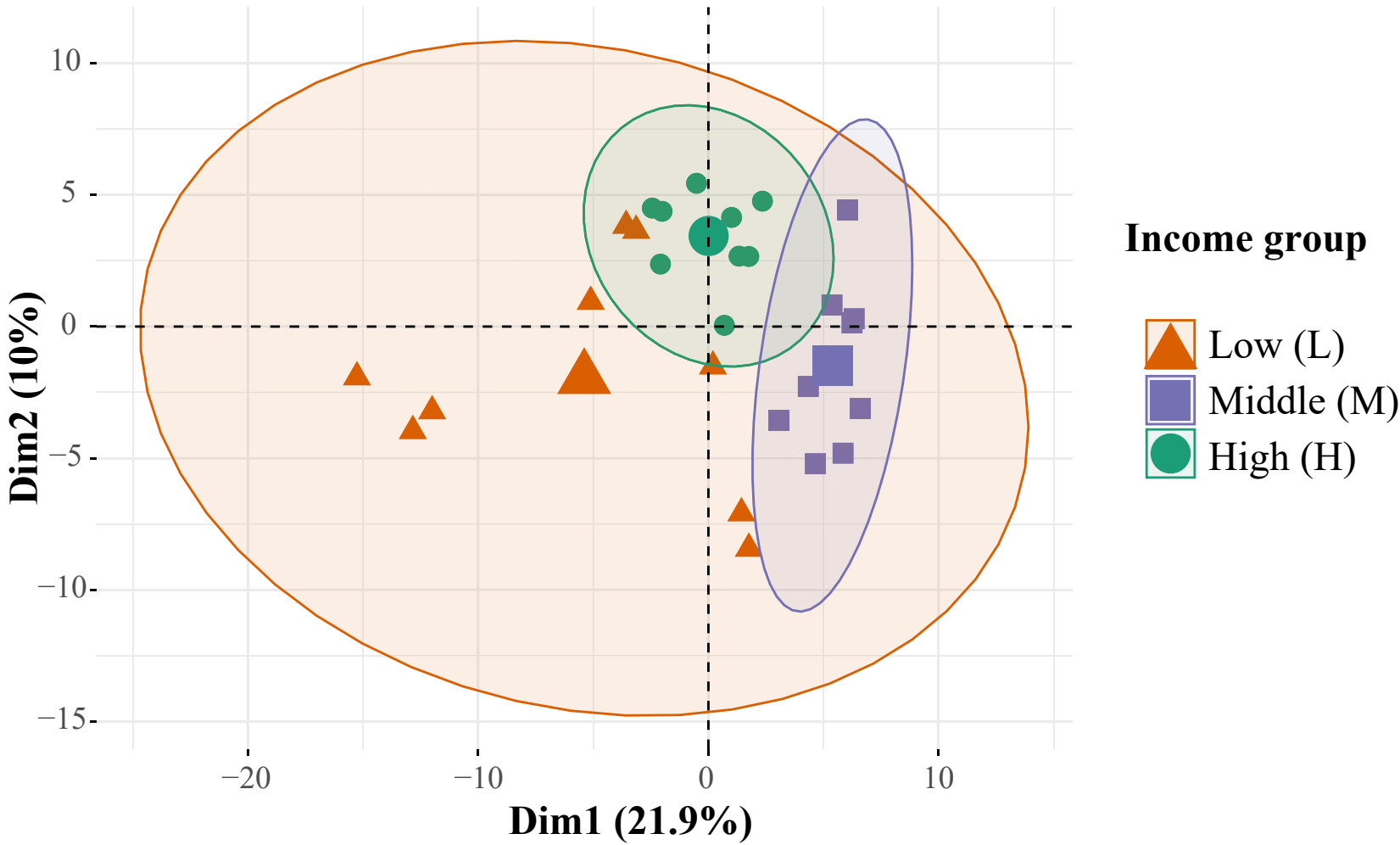

B

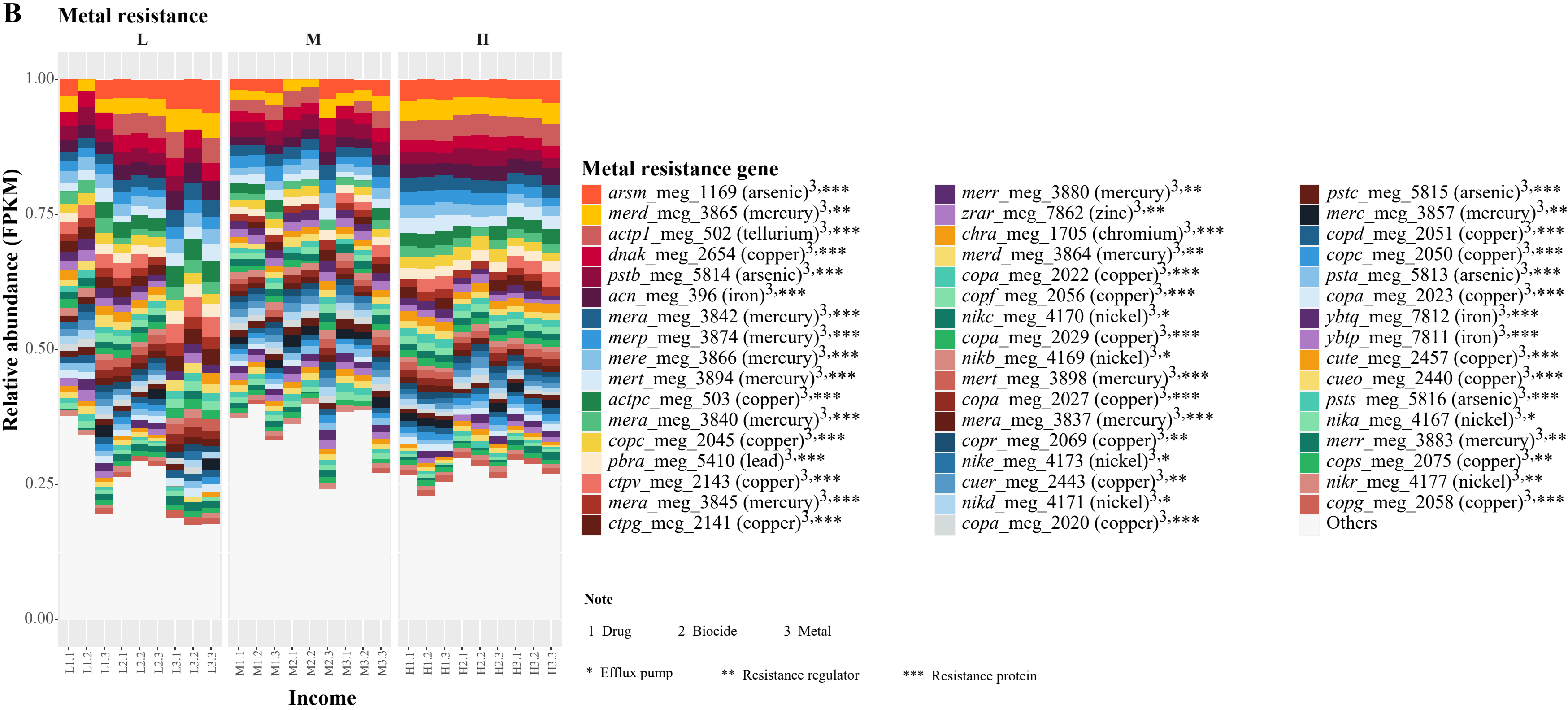

C

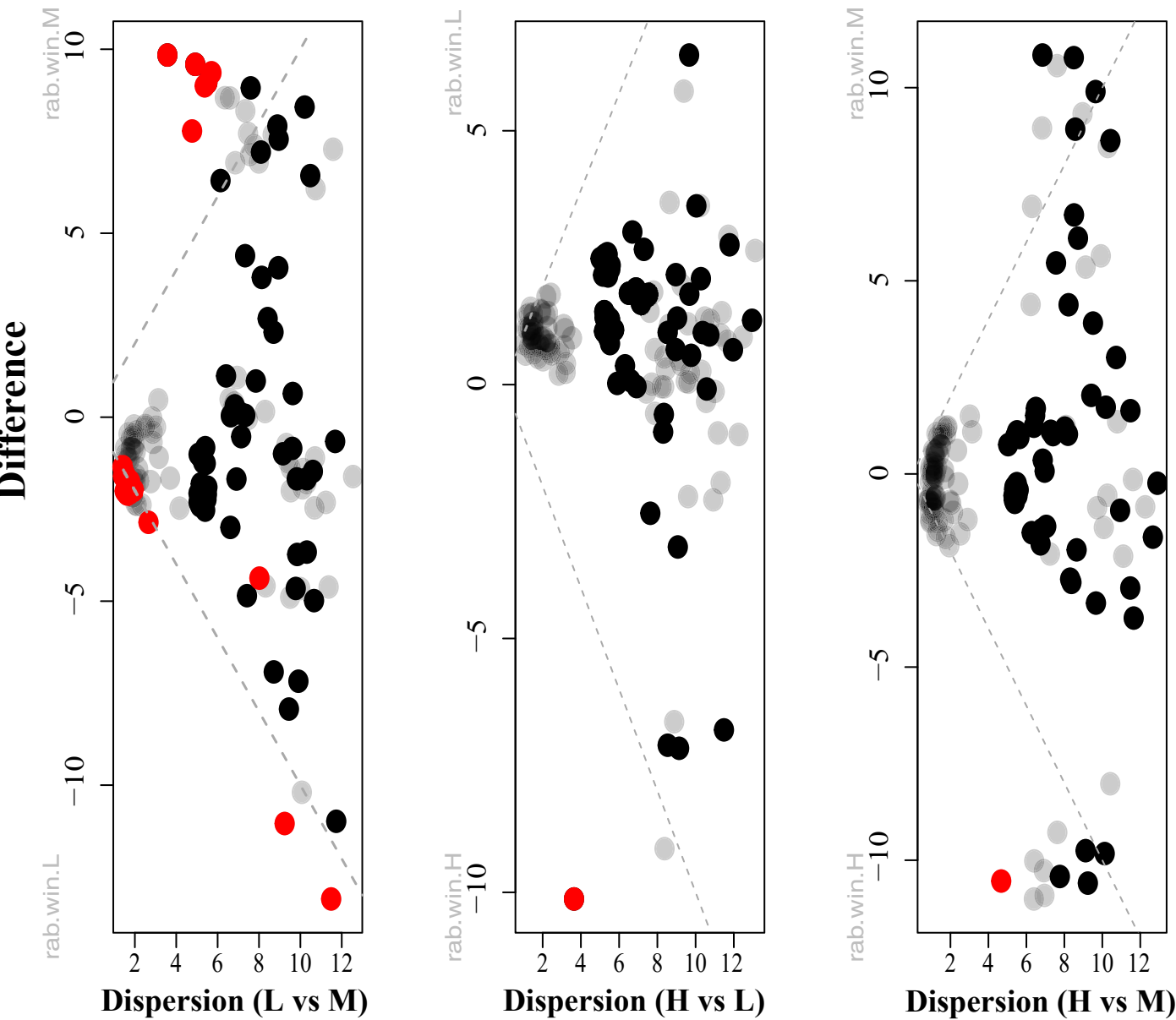

D

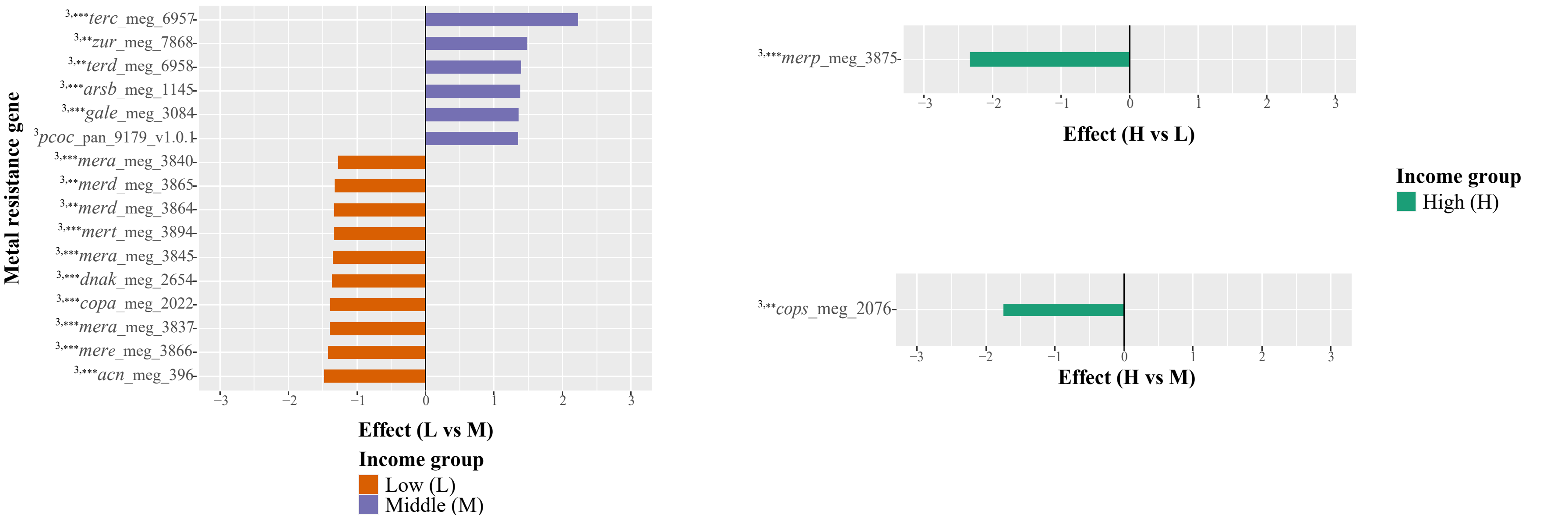

Supplement: Supplementary file 7 [file Data_Sheet_5.PDF]

A

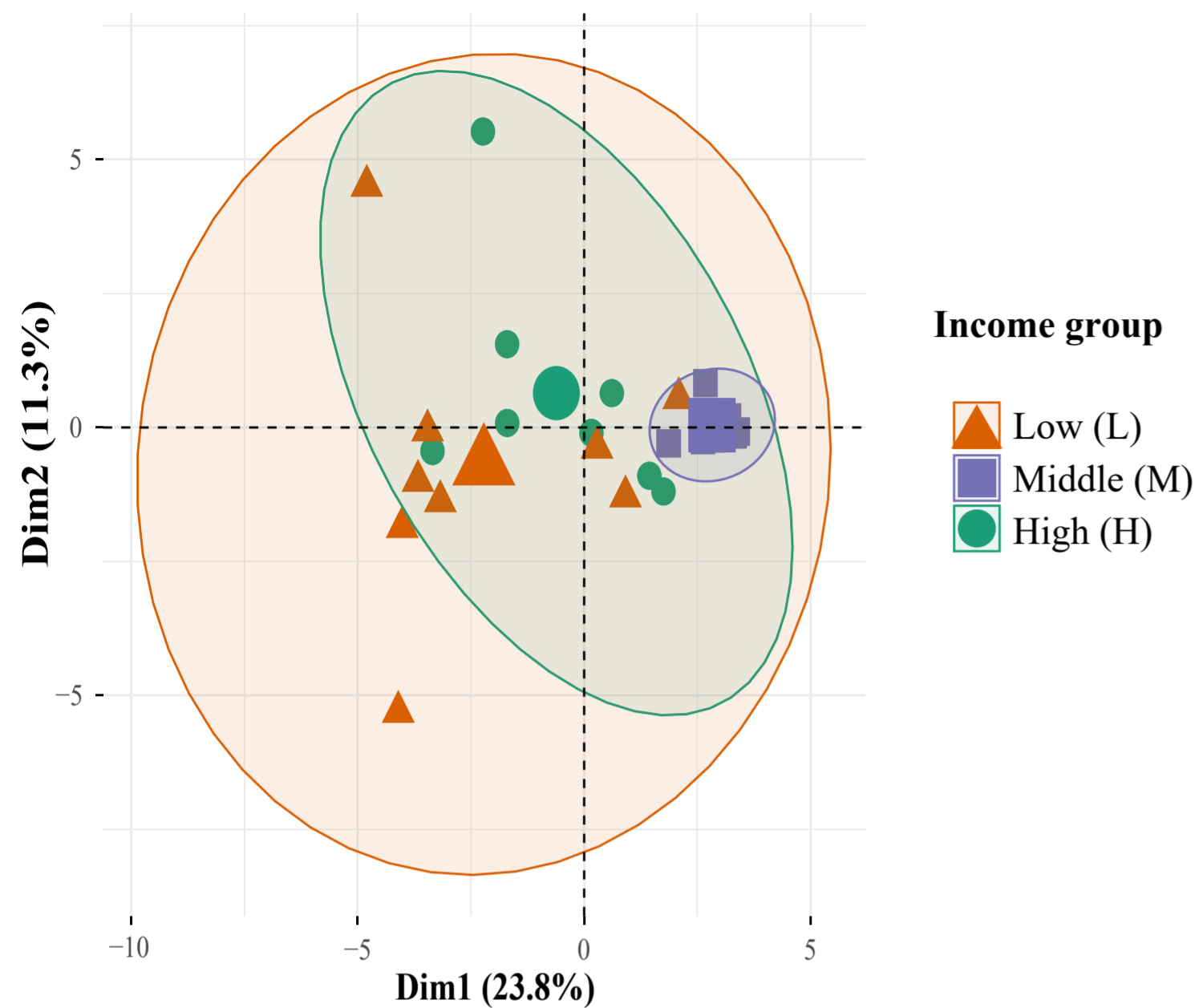

B

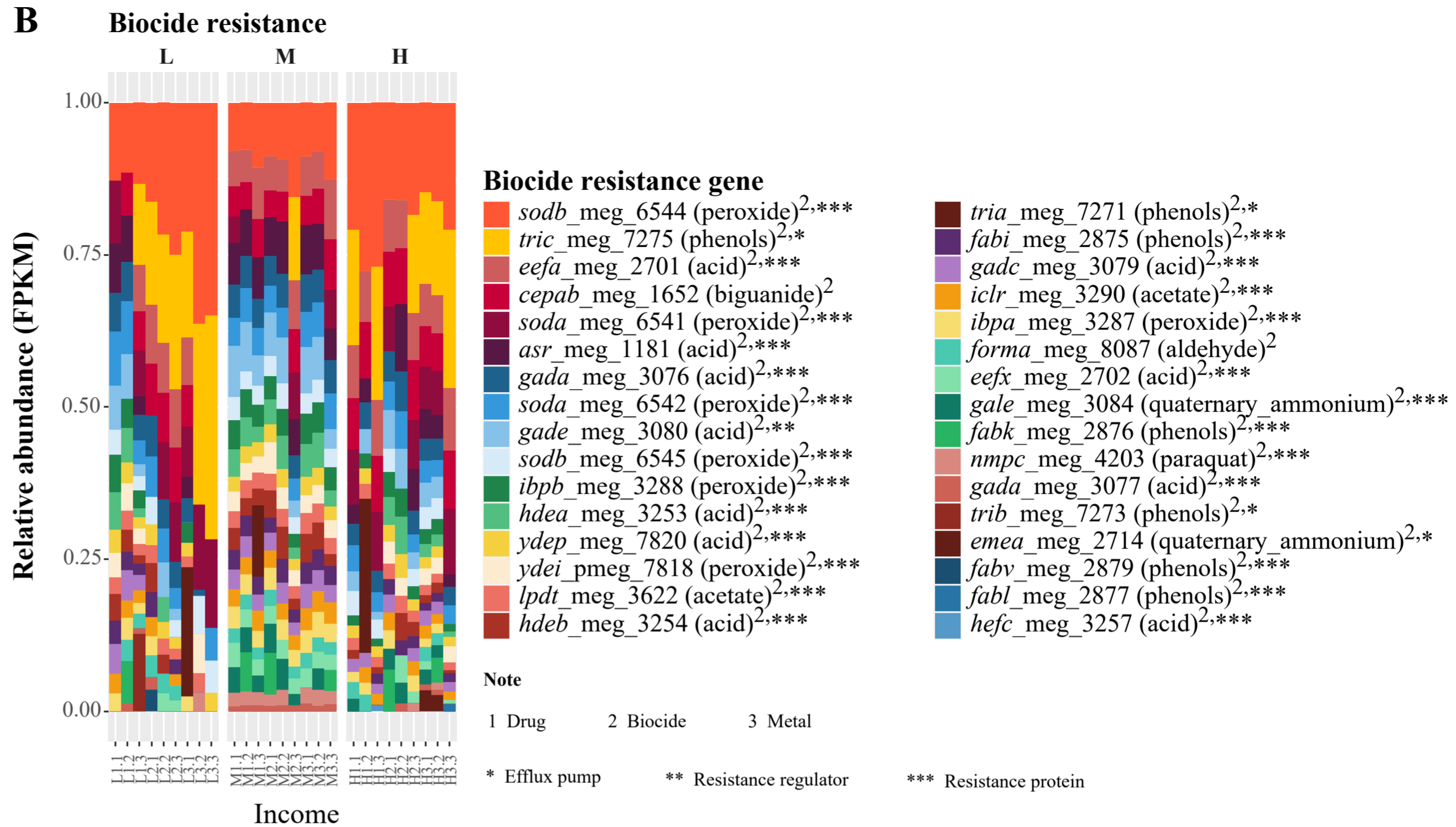

C

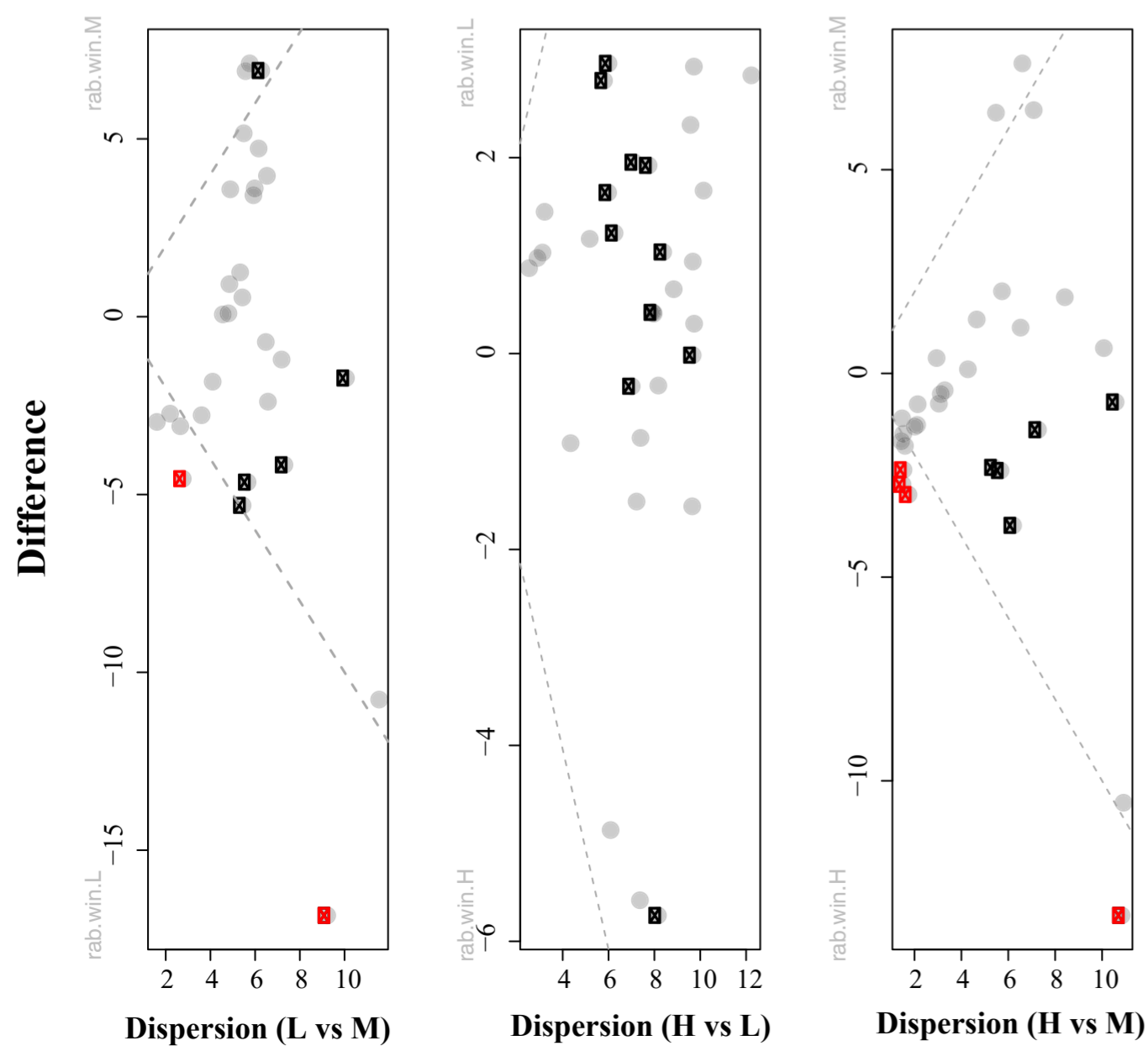

D

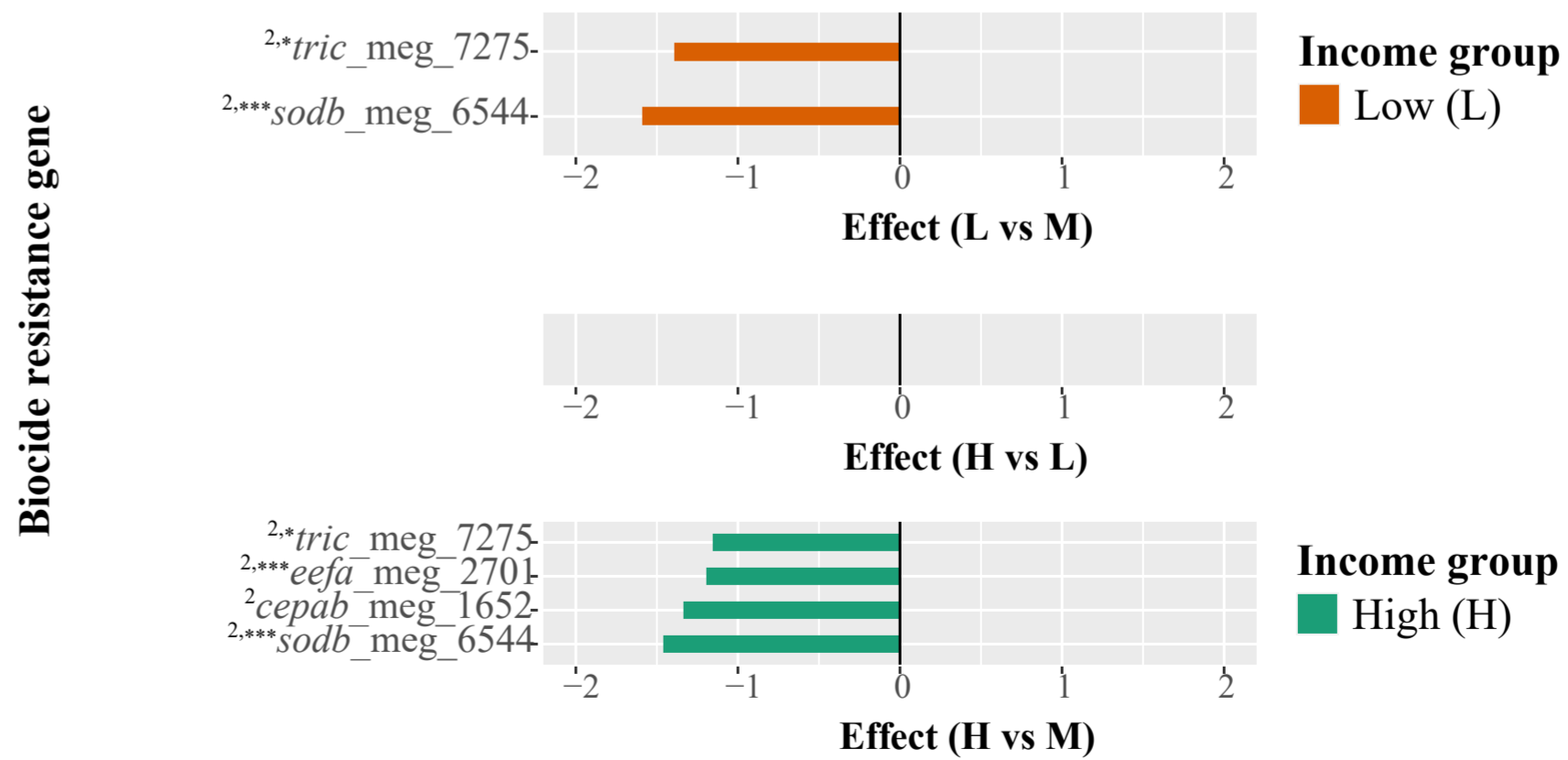

Supplement: Supplementary file 8 [file Data_Sheet_6.PDF]

A

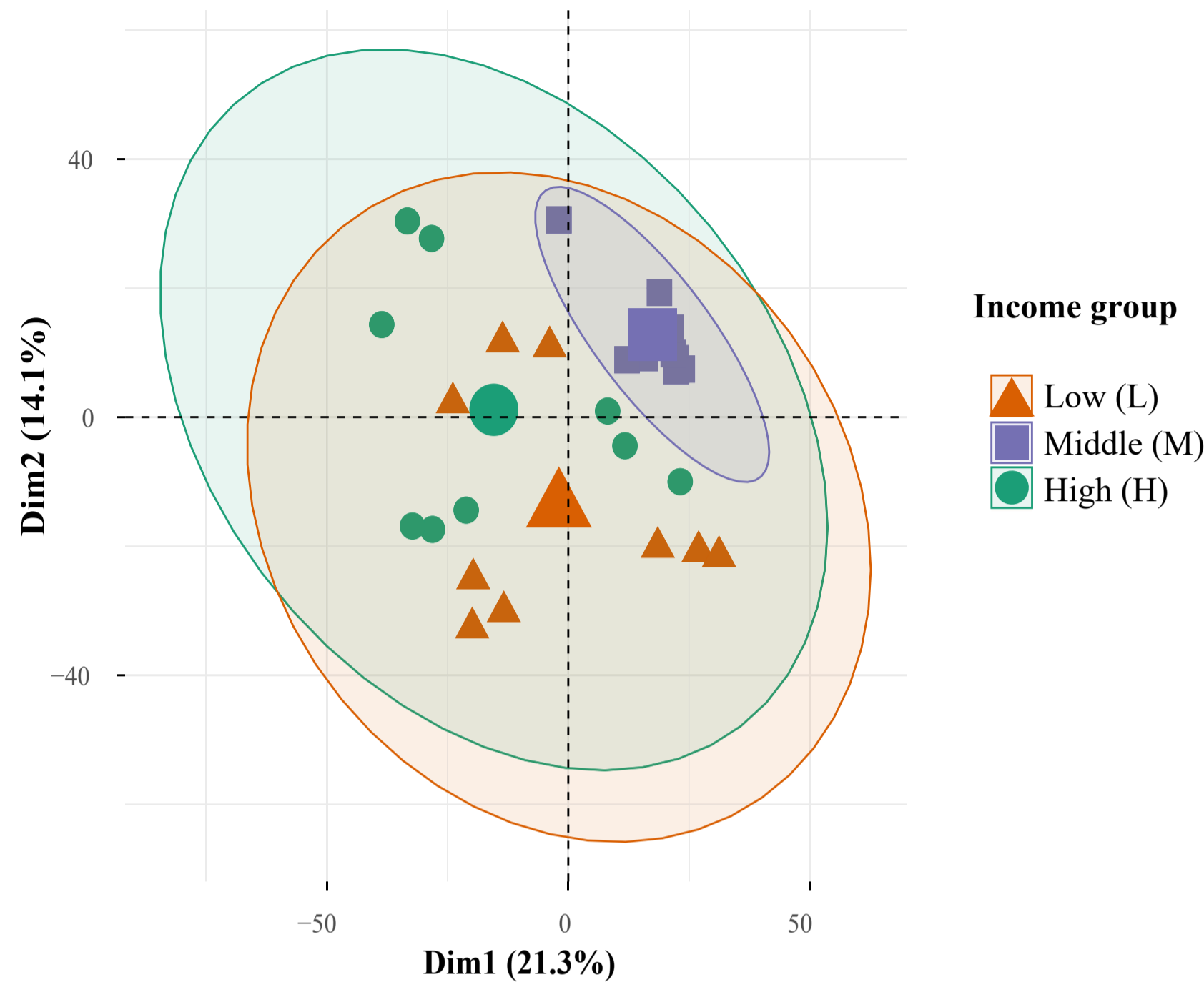

B

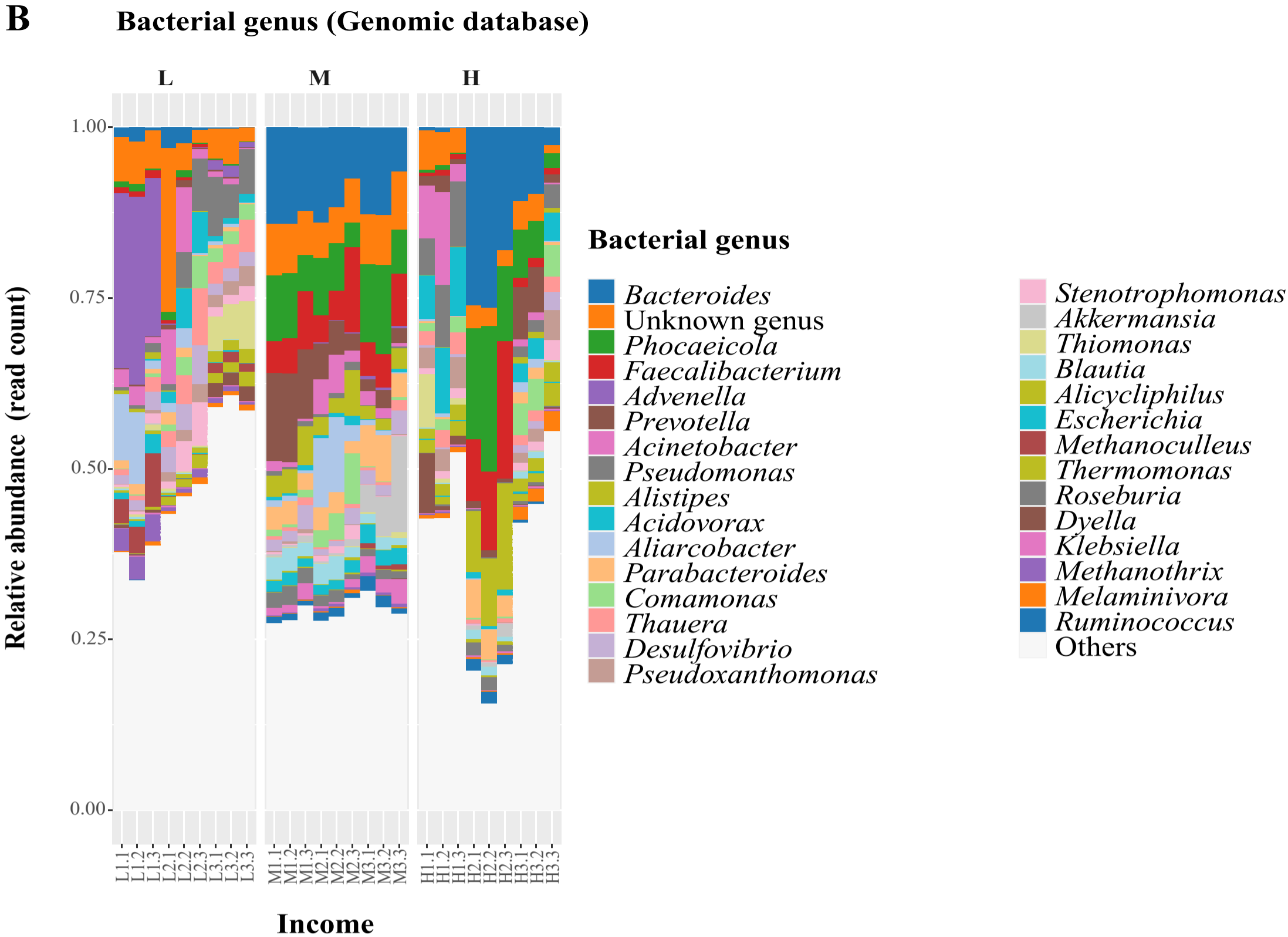

C

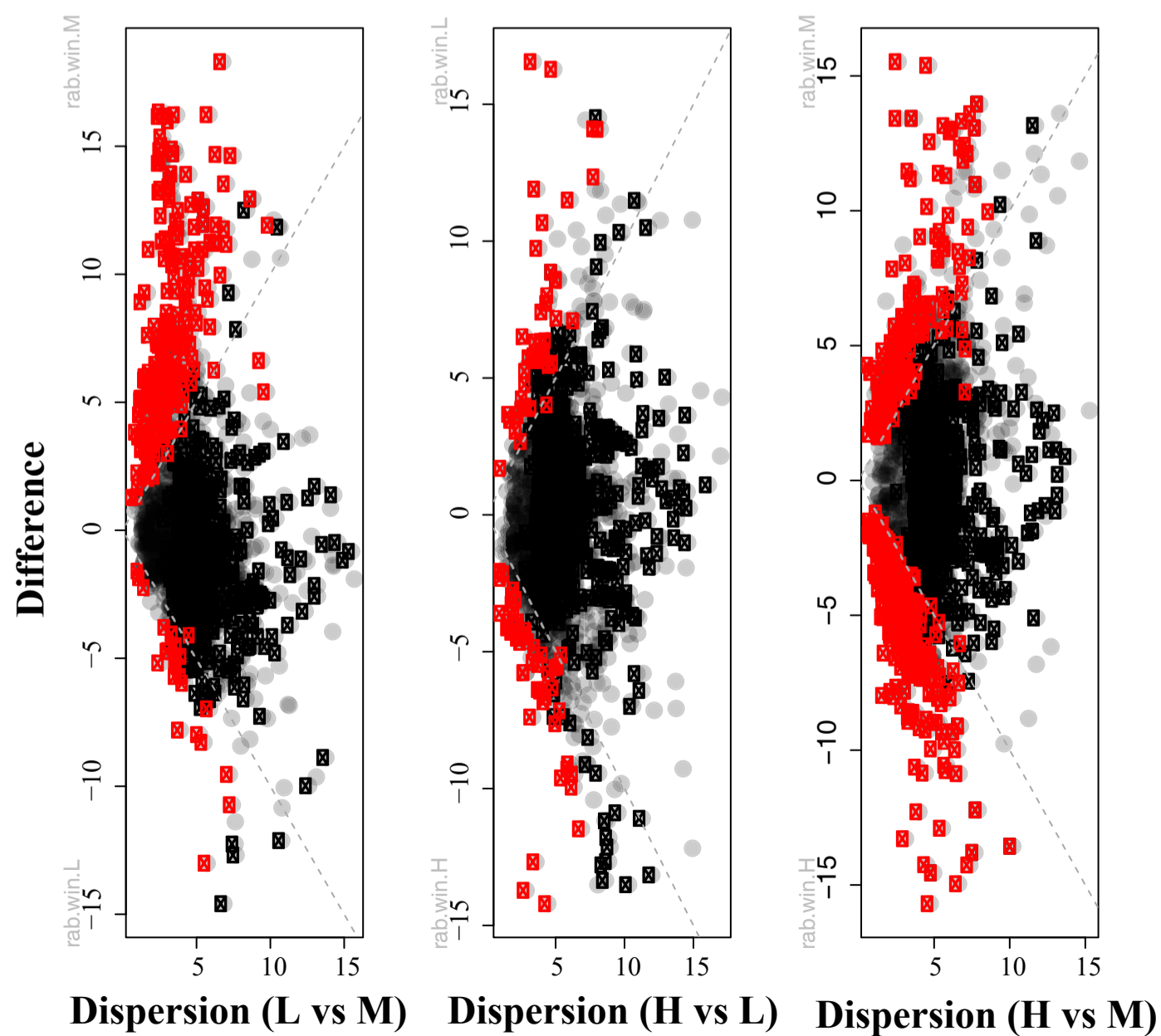

D

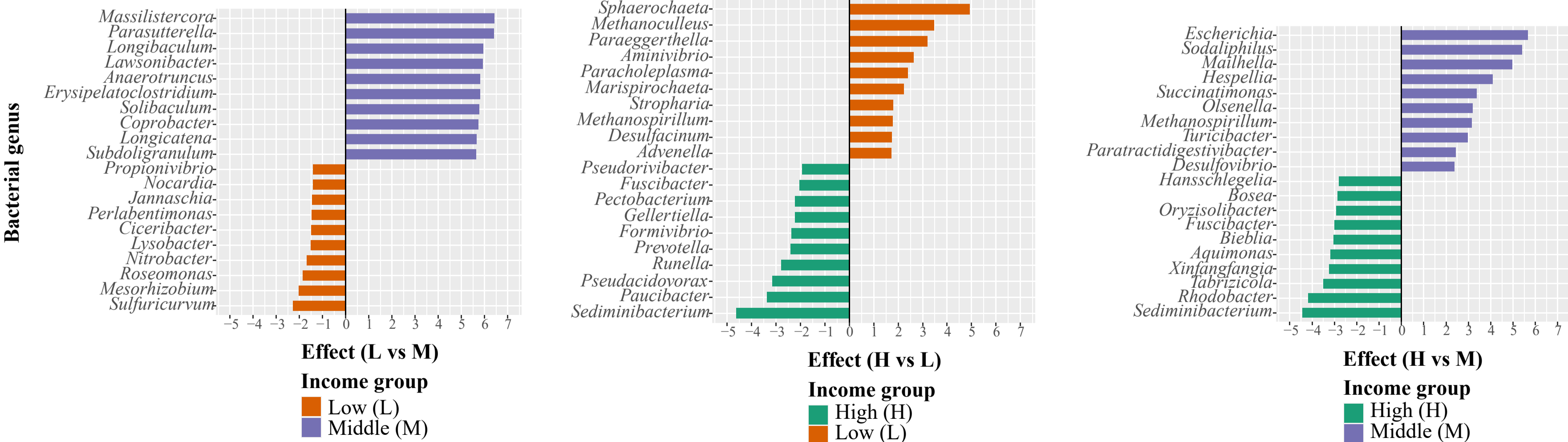

Supplement: Supplementary file 9 [file Data_Sheet_7.PDF]
