# Supplementary material for: Comparative resistome from toilet waste in three different income areas, Bangkok, Thailand
Source: Front Microbiol. 2026 Mar 25;17:1790551. doi: 10.3389/fmicb.2026.1790551 (PMC13057367; doi:10.3389/fmicb.2026.1790551)
Supplement: Supplementary file 4 [file Data_Sheet_2.PDF]

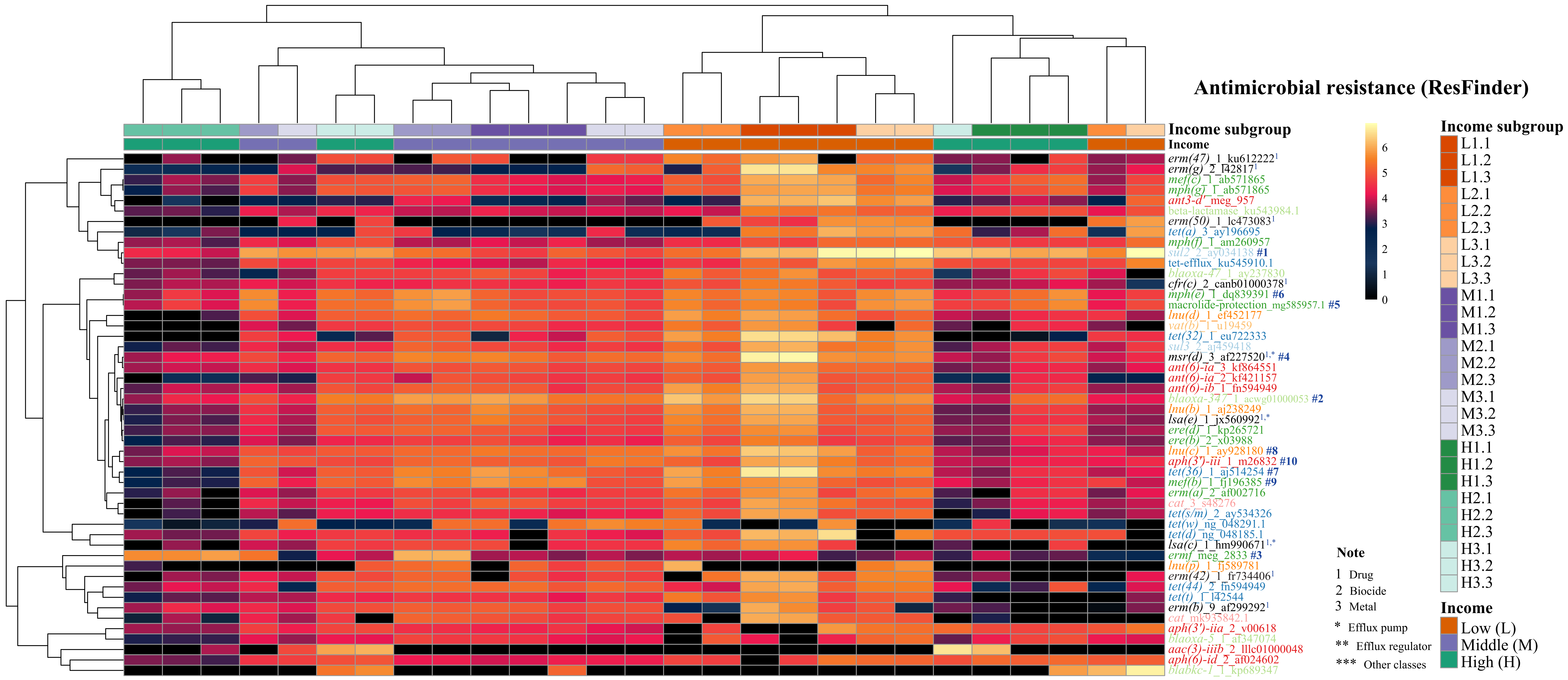

Antimicrobial resistance (ResFinder)
Income subgroup
Income
erm(47)_1_ku612222 1
erm(g)_2_l42817 1
mef(c)_1_ab571865
mph(g)_1_ab571865
ant3-d'_meg_957
beta-lactamase_ku543984.1
erm(50)_1_lc473083 1
tet(a)_3_ay196695
mph(f)_1_am260957
sul2_2_ay034138 #1
tet-efflux_ku545910.1
blaoxa-47_1_ay237830
cfr(c)_2_canb01000378 1
mph(e)_1_dq839391 #6
macrolide-protection_mg585957.1 #5
lnu(d)_1_ef452177
vat(b)_1_u19459
tet(32)_1_eu722333
sul3_2_aj459418
msr(d)_3_af227520 1,* #4
ant(6)-ia_3_kf864551
ant(6)-ia_2_kf421157
ant(6)-ib_1_fn594949
blaoxa-347_1_acwg01000053 #2
lnu(b)_1_aj238249
lsa(e)_1_jx560992 1,*
ere(d)_1_kp265721
ere(b)_2_x03988
lnu(c)_1_ay928180 #8
aph(3')-iii_1_m26832 #10
tet(36)_1_aj514254 #7
mef(b)_1_fj196385 #9
erm(a)_2_af002716
cat_3_s48276
tet(s/m)_2_ay534326
tet(w)_ng_048291.1
tet(d)_ng_048185.1
lsa(c)_1_hm990671 1,*
ermf_meg_2833 #3
lnu(p)_1_fj589781
erm(42)_1_fr734406 1
tet(44)_2_fn594949
tet(t)_1_l42544
erm(b)_9_af299292 1
cat_mk935842.1
aph(3')-iia_2_v00618
blaoxa-5_1_af347074
aac(3)-iiib_2_lllc01000048
aph(6)-id_2_af024602
blabkc-1_1_kp689347
6
5
4
3
2
1
0
Note
1 Drug
2 Biocide
3 Metal
* Efflux pump
** Efflux regulator
*** Other classes
Income subgroup
L1.1
L1.2
L1.3
L2.1
L2.2
L2.3
L3.1
L3.2
L3.3
M1.1
M1.2
M1.3
M2.1
M2.2
M2.3
M3.1
M3.2
M3.3
H1.1
H1.2
H1.3
H2.1
H2.2
H2.3
H3.1
H3.2
H3.3
Income
Low (L)
Middle (M)
High (H)
