# Supplementary material for: Comparative resistome from toilet waste in three different income areas, Bangkok, Thailand
Source: Front Microbiol. 2026 Mar 25;17:1790551. doi: 10.3389/fmicb.2026.1790551 (PMC13057367; doi:10.3389/fmicb.2026.1790551)
Supplement: Supplementary file 10 [file Data_Sheet_8.PDF]

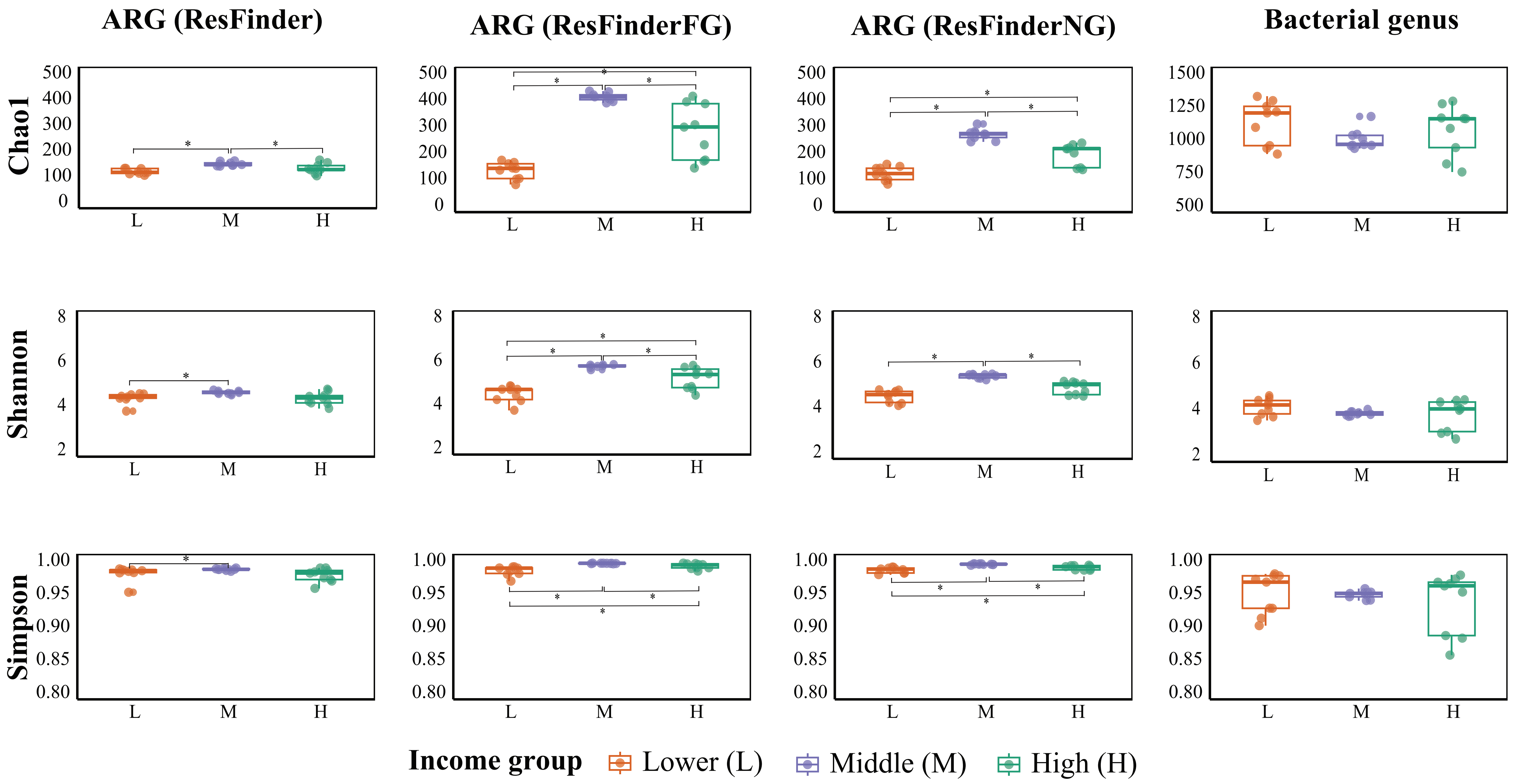

ARG (ResFinder)
ARG (ResFinderFG)
ARG (ResFinderNG)
Bacterial genus
Chao1
Shannon
Simpson
L
M
H
Income group
Lower (L)
Middle (M)
High (H)
